# Enzyme Synergy in Transient Clusters of Endo- and Exocellulase Enables a Multilayer Mode of Processive Depolymerization of Cellulose

**DOI:** 10.1021/acscatal.2c02377

**Published:** 2022-08-24

**Authors:** Krisztina Zajki-Zechmeister, Manuel Eibinger, Bernd Nidetzky

**Affiliations:** † Institute of Biotechnology and Biochemical Engineering, Graz University of Technology, Petersgasse 10-12/1, 8010 Graz, Austria; ‡ Austrian Centre of Industrial Biotechnology, Petersgasse 14, 8010 Graz, Austria

**Keywords:** polysaccharide materials, cellulose, cellulase, endo−exo enzyme synergy, transient clusters, processive degradation

## Abstract

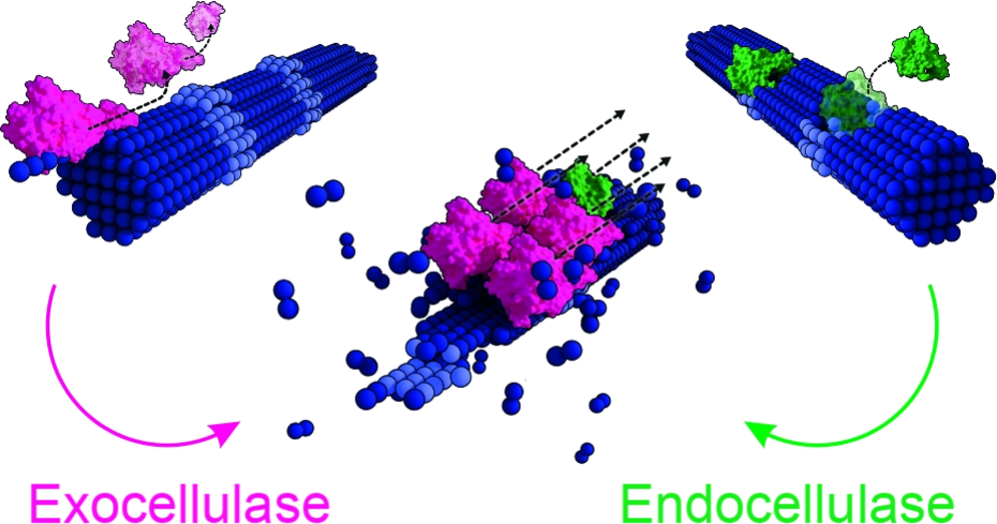

Biological degradation of cellulosic materials relies on the molecular-mechanistic principle that internally chain-cleaving endocellulases work synergistically with chain end-cleaving exocellulases in polysaccharide chain depolymerization. How endo–exo synergy becomes effective in the deconstruction of a solid substrate that presents cellulose chains assembled into crystalline material is an open question of the mechanism, with immediate implications on the bioconversion efficiency of cellulases. Here, based on single-molecule evidence from real-time atomic force microscopy, we discover that endo- and exocellulases engage in the formation of transient clusters of typically three to four enzymes at the cellulose surface. The clusters form specifically at regular domains of crystalline cellulose microfibrils that feature molecular defects in the polysaccharide chain organization. The dynamics of cluster formation correlates with substrate degradation through a multilayer-processive mode of chain depolymerization, overall leading to the directed ablation of single microfibrils from the cellulose surface. Each multilayer-processive step involves the spatiotemporally coordinated and mechanistically concerted activity of the endo- and exocellulases in close proximity. Mechanistically, the cooperativity with the endocellulase enables the exocellulase to pass through its processive cycles ∼100-fold faster than when acting alone. Our results suggest an advanced paradigm of efficient multienzymatic degradation of structurally organized polymer materials by endo–exo synergetic chain depolymerization.

## Introduction

Major polysaccharides in nature, such as those built from the common sugar d-glucose (e.g., cellulose, starch), are important biomaterials and represent global reserves of carbohydrate.^1,2^ Many organisms use them as substrates for life. Natural utilization involves coordinated systems of multiple enzymes of distinct specificity, and showing synergetic function, in polysaccharide chain depolymerization.^3,4^

**Figure 1:**
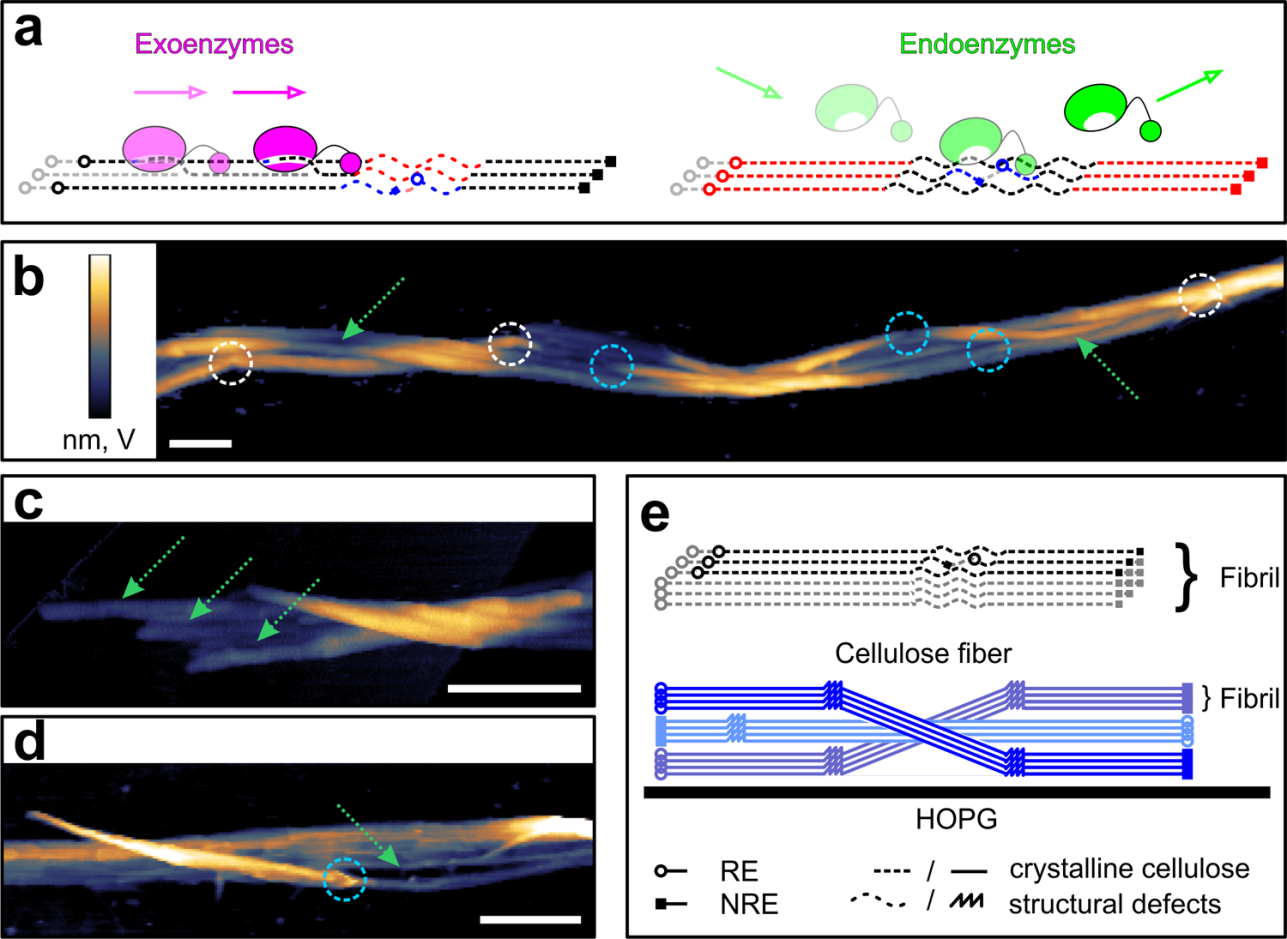
Schematic representation of the cellulose chain depolymerization by endo- and exocellulases, and the substrate nanoarchitecture of bacterial cellulose fibers used in single-molecule enzyme studies. (a) Processive depolymerization of crystalline cellulose by chain end-cleaving exoenzymes (magenta) and degradation of less ordered cellulose nanodomains by internal chain cleavages of endoenzymes (green). Exoenzymes with specificity for the reducing (RE, open circles) and the nonreducing chain end (NRE, full squares) are known. RE cleavage is shown. (b–d) Atomic force microscopy (AFM) height images of cellulose fibers adsorbed on the highly oriented pyrolytic graphite wafer used in experiments. (b) Individual cellulose fibrils (arrows) and structural defects in the polysaccharide chain organization (circles) are highlighted. Defects in crystalline material organization include “loose” fibril ends (light blue) and kinks (white). (c) Terminal region of a cellulose fiber with multiple isolated fibrils unwound from the fibril bundle (arrows). (d) Exemplary fiber with a disentangled fibril (arrow) featuring “loose” ends (light blue circle) on the surface. (e) Schematic representation of the bottom-up nanoarchitecture of the bacterial cellulose fiber. Multiple fibrils made from repeating units of cellobiose are twined together, forming the fiber. The false color scales used throughout the images are shown in (b) and should be read from bottom (minimum) to top (maximum). Height (nm) ranges were 33 nm (b), 20 nm (c), and 15 nm (d). Scale bars are 100 nm.

A universal kind of enzyme synergy in polysaccharide degradation is that between internally chain-cleaving endoenzymes and processively chain end-cleaving exoenzymes (Figure 1a).^4−8^ Endo–exo synergy is intuitively explained by a reciprocal generation of substrate sites between the two types of enzyme.^4,6,9,10^ Each endo cleavage releases new chain ends for the exoenzyme. Exo-type processive chain depolymerization uncovers new internal sites for the endoenzyme within the polysaccharide network of substrate material.^10−12^ The cooperative interplay between endo- and exoenzymes results in a degradation rate that can be several-fold enhanced over the sum of the individual enzyme rates.^10,13,14^ When maintained over the relevant course of substrate degradation, it can additionally become a significant factor of the product yield in polysaccharide bioconversion processes.^15,16^ As a molecular-mechanistic principle, therefore, endo–exo synergy has high fundamental but also practical importance.

It follows from the proposed mechanism of cooperative action that the synergistic effect generated by a mixture of endo- and exoenzyme depends on the relative proportion of the two components.^6,12,17−19^ The immediate ramification for enzyme development, that endo–exo composition represents a key engineering target in optimizing the overall specific activity, has had strong appeal in the field of cellulose bioconversion.^19−21^ Due to the significant costs incurred from the high enzyme loadings required in the process,^7,22^ an efficiency-enhanced cellulase cocktail promises considerable financial leverage towards commercial viability.^23,24^ However, the aggregate evidence from a large number of studies of endo–exo synergy in cellulose hydrolysis^6,10−12,15,17,25−29^ is fundamentally at variance with expectation for the canonical mechanism of substrate site generation. In particular, the finding that endo- and exocellulase synergize effectively even when the substrate is available in large excess for both enzymes^6,17,27−29^ points to the necessity to overhaul the apparently well-accepted mechanistic thinking.

Discovery of the current study that endo–exo synergy among cellulases involves transient clusters of the cooperatively acting enzymes provides answer to a long outstanding question. Using fast atomic force microscopy (AFM) in liquid environment to monitor the enzymatic degradation of crystalline cellulose fibers in real time, we were able to characterize the synergetic activity of endo- and exocellulase at single-molecule resolution. We show that transient clusters of endo- and exocellulase, formed at specific sites of the cellulose surface, are enabled to a previously unrecognized, highly efficient “multilayer mode” of processive chain depolymerization. The mechanistic basis of endo–exo synergy is thus revealed: cooperativity with the endocellulase makes the exocellulase move between its processive catalytic cycles much faster (≥100-fold) than when acting alone. The evidence presented establishes a new mechanistic paradigm of how cellulase systems exploit endo–exo synergy to gain efficiency in deconstructing cellulose materials.

## Results

### Multilayer Mode of Processive Degradation of Cellulose Chains

The experimental framework of our earlier AFM study was used,^30^ except that measurements were performed at ∼10-fold higher temporal resolution, with up to 2 frames/s recorded. AFM observations were made in Tapping Mode (see the Atomic Force Microscopy section in the Supporting Information for details) in temperature-controlled liquid environment (35 °C). Single fibers of crystalline bacterial cellulose adsorbed on the surface of highly oriented pyrolytic graphite were analyzed (Figure 1b–e, see Preparation of Single Bacterial Cellulose Fibers in the Supporting Information). The cellulases used were from the wood-degrading fungus *Trichoderma reesei*. They represent a complete enzyme system for the hydrolytic solubilization of cellulose and comprise two major exocellulases (Cel7A, Cel6B) as well as several endocellulases, most prominently Cel7B and Cel5A.^4,31,32^ Additionally, β-glucosidases, which are not directly involved in the depolymerization of cellulose, are present for the conversion of soluble oligosaccharides (mostly cellobiose) to glucose.^4^ As previously shown, the enzymes acting on the cellulosic substrate degrade the cellulose fiber via directed ablation of surface-exposed microfibrils.^30^ The exocellulase Cel7A which degrades cellulose chains via processive chain cleavages from the reducing end (Figure 1a–c)^4,33,34^ is responsible for the observed directionality of microfibril deconstruction by the *T. reesei* cellulases.^30^

**Figure 2:**
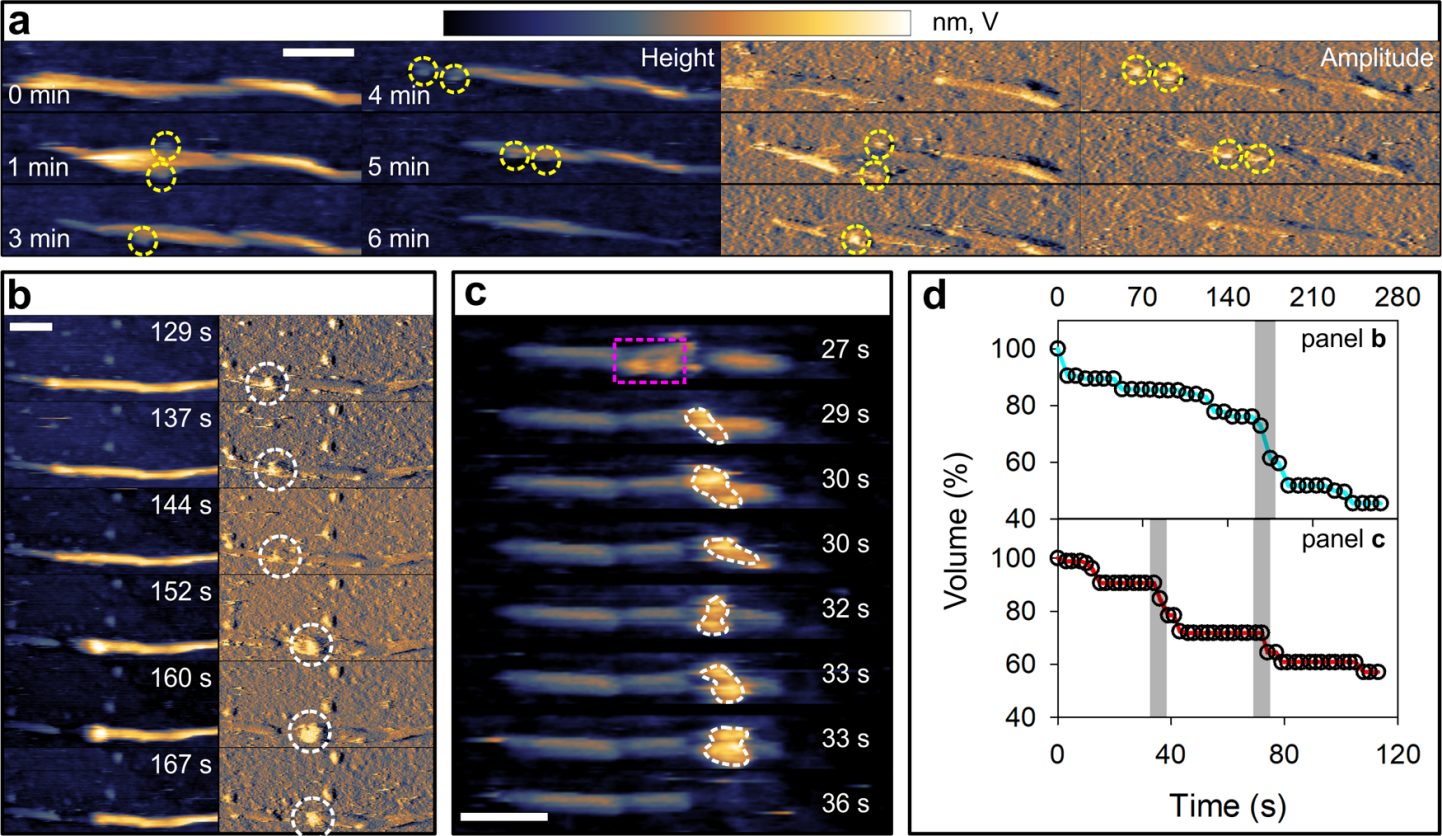
Cellulose fiber deconstruction by the whole cellulase system of *T. reesei* involves the unique activity of transient clusters of multiple enzymes formed at the cellulose surface. (a) Sample height (left) and amplitude (right) images from a fast AFM observation of single cellulases (yellow circle) sliding along a cellulose fibril. The degradation occurs along a preferred direction via shortening and thinning. (b, c) AFM height and amplitude images taken from the high-speed Movie S1, showing multilayer degradation of entire fibril parts through a discretely discontinuous series of processive steps. Enzyme clusters are enveloped in white. In (c), an incipient enzyme cluster is framed in magenta. (d) Time course of volume loss during fiber degradation. Time periods of rapid volume loss are associated with the activity of enzyme clusters and are highlighted in gray. Scale bars are 50 nm. The false color scale used throughout the images is shown in (a) and should be read from left (minimum) to right (maximum). Height (nm) and amplitude (V) ranges were 10 nm/60 V (a), 12 nm/27 V (b), and 6 nm (c).

Using AFM measurements with high time resolution (up to ∼2 fps) (Figure 2 and Movie S1), we here observed that the enzymatic fibril degradation happens through two concurrently operating modes. One mode consists in the continuous removal of small amounts of the fiber volume, which may indeed reflect the ablation of single surface layers of cellulose one at a time. To achieve this, many single molecules of cellulase are seen in dynamic interaction with the cellulose surface (Figure 2a and Movie S1). Enzymes appear (adsorb) on, and disappear (desorb) from, the cellulose fiber analyzed, and several of them move on the cellulose surface in a preferred direction, consistent with earlier visualization studies of processive cellulases including Cel7A.^35−37^ The other mode is fundamentally different, for it consists in a multilayer degradation of entire fibrils. It proceeds through a discretely discontinuous series of processive steps (Figure 2b,c and Movies S1 and S2). Each step is represented by a rapid loss of fiber volume in the time course of cellulose degradation (Figure 2d). The observed multilayer mode of cellulose degradation was puzzling mechanistically. Its appearance contrasts with the expectation from the widely held notion that cellulases operate as a dispersed ensemble of individual, independently acting enzymes.^4,38,39^ Working solely in this “ensemble style”, the cellulases would be restricted to degrading the substrate in the lateral dimension via single-layer cellulose chain ablation. How cellulases acquire the distinct, transversally directed component of their degradation of the cellulose fibrils required explanation.

### Transient Clusters of Cellulase Involved in Multilayer-Processive Degradation

**Figure 3:**
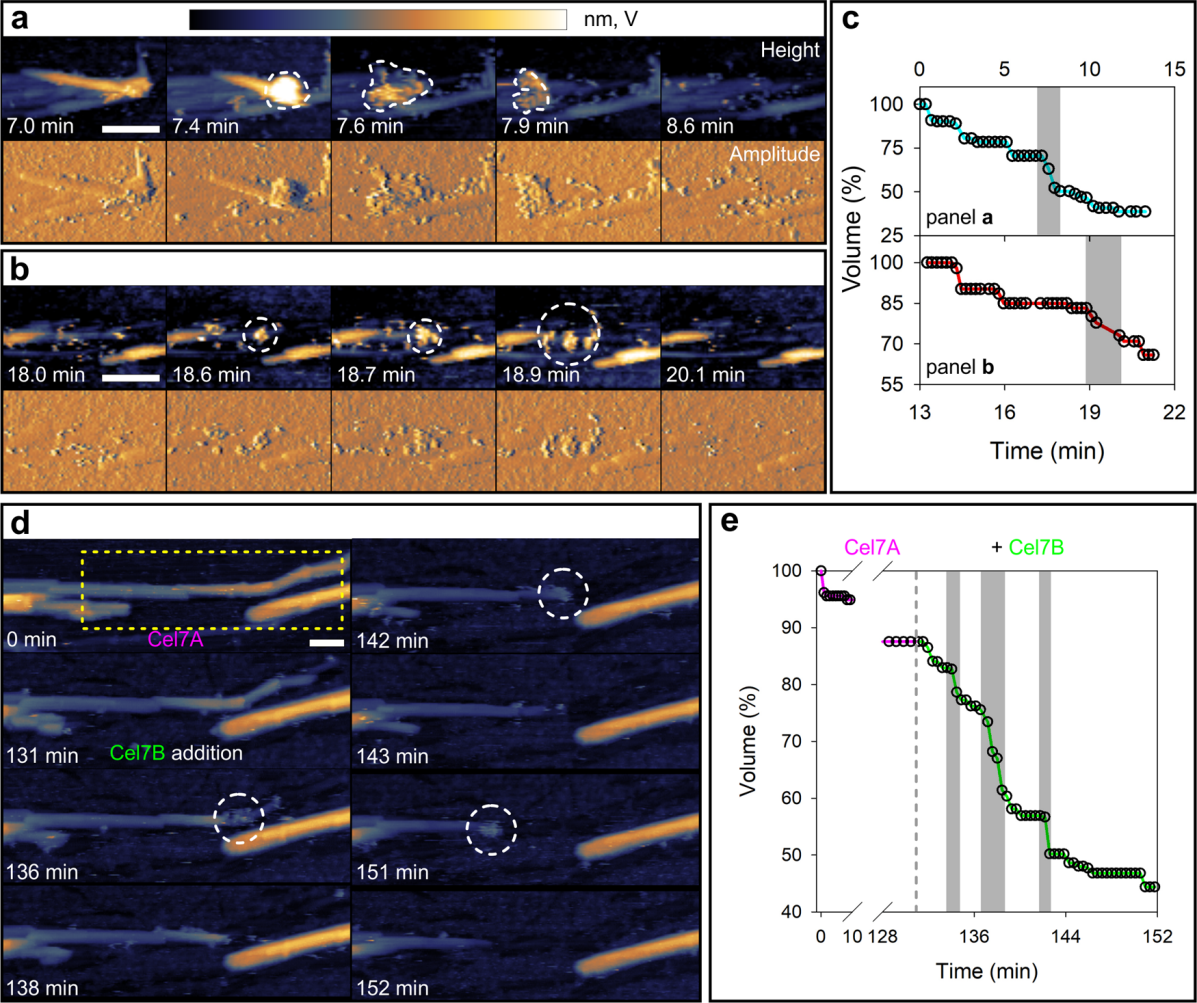
Dynamics of the molecular assembly of cellulase clusters and its relationship with cellulose degradation. (a, b) Height (left) and amplitude (right) images from AFM observations of transient enzyme clusters (encircled in white) degrading cellulose fibrils. The enzyme cluster in (a) (∼6 enzymes) is larger than that in (b) (∼3 enzymes). (c) Time course of volume loss during fiber degradation. Time periods of rapid volume loss associated with enzyme cluster activity are highlighted in gray. (d) AFM results showing cellulose fibril deconstruction by Cel7A acting alone and in combination with the endoglucanase Cel7B. The image strip visualizes the fibril degradation and shows enzyme cluster formation (white circle). The full sequence is in Movie S4, which reveals multiple enzyme clusters formed, but only when Cel7A and Cel7B both are present. (e) Time course showing the loss in fibril volume associated with Cel7A acting alone (magenta) and in combination with Cel7B (green) in the yellow rectangle. Prominent degradation events due to the activity of an enzyme cluster are highlighted in gray. Scale bars are 50 nm. The false color scale used throughout the images is shown in (a) and should be read from left (minimum) to right (maximum). Height (nm) and amplitude (V) ranges were 11 nm/48 V (a), 5 nm/47 V (b), and 16 nm (d).

We analyzed ∼100 separate events of multilayer cellulose degradation in detail (see AFM analysis—Cluster Size, Speed and Degradation in the Supporting Information). Results show that each processive step involves at its start the assembly of multiple cellulases in close proximity (Figure 2c and Movie S2). Processive degradation is accompanied by joint lateral movement of the cellulases and is terminated by enzyme cluster disengagement (Figure 2b,c and Movies S1 and S2). The enzyme cluster formation involves notable regularities that suggest recognition of distinct regions of cellulose surface as “cluster initiation sites”. The clusters are formed preferably at internal nanodomains of the microfibrils that feature defects (Figure 2b,c and Movies S1 and S2) in the crystalline organization of the cellulose chains. Additionally, they are also found at the fibril ends (Figure 3a,b). Here, it is instructive to consider the characteristic nanoarchitecture of the cellulose microfibrils. As shown in our earlier study,^30^ nanodomains of different (high/low) structural orders alternate in a somewhat regular fashion along the fibril length. The cellulase clusters are formed specifically at the structurally less ordered nanodomains (nanoscale “defects” of crystalline material) or “loose” fibril ends.

To rule out that the cellulase cluster formation was merely an effect of the enzyme loading used, we performed AFM experiments at a 5-fold reduced enzyme concentration (20 μg/mL) and the clusters were still present (Figure S1). The result suggests that the enzyme cluster formation is an intrinsic molecular characteristic of cellulase activity. We also show that enzyme clusters represent an important factor of cellulase efficiency in cellulose degradation. Analyzing fiber parts of a volume of up to ∼5000 nm^3^, we find that fibril degradation by enzyme clusters contributes substantially (up to ∼50%, Figures 2d and 3c) to the overall fiber deconstruction.

### Transient Clusters of Cellulase Involve Endo and Exo Chain-Cleaving Activities in Dynamic Assembly

To clarify the molecular origin of cellulase activity in dynamic multienzyme clusters, we performed AFM experiments using individual cellulases as isolated enzyme preparations. From the observed “substrate specificity” involved in cellulase cluster formation, we speculated that both endo- and exocellulases are involved in the process. Endocellulases are widely believed to attack surface sites of crystalline cellulose that exhibit defects in the molecular organization of polysaccharide chains.^30,40−42^ We here used Cel7A (the major exocellulase of the *T. reesei* cellulase system)^4,10,43,44^ and examined it in combination with Cel7B (a representative endocellulase of the system).^4,26^ Applied as single enzymes, neither Cel7A (Figures S2 and S3) nor Cel7B (Figure S4) forms clusters on the cellulose surface, even when used at significantly (100-fold, Figure S3) elevated protein concentrations (see Application of Cel7A as Single Enzyme at Different Concentrations in the Supporting Information). Fiber degradation by Cel7A is continuous, with steps of rapid volume loss clearly lacking (Figure 3d,e and first part of Movie S4). Cel7B alone does not degrade the fiber in a notable degree (Figure S4). However, when Cel7B is added to a Cel7A reaction, enzyme clusters appear in large numbers all over the cellulose surface, at attack sites structurally analogous to the ones identified with the whole cellulase mixture (Figure 3e and second part of Movie S4). The fiber deconstruction thus becomes dominated by the processive steps of multilayer fibril degradation promoted by the enzyme clusters (Figure 3e and Movie S4).

To test the classical mechanistic interpretation of endo–exo synergy that the endocellulase prepares substrate for the exocellulase,^4,10,14,45^ we incubated the cellulose with Cel7B, removed the enzyme, and used the pretreated fibers as substrate of Cel7A. We show that Cel7A clusters do not form on the Cel7B-treated cellulose (see Preparation of Cel7B-Treated Cellulose Fibers and Their Subsequent Degradation by Cel7A in the Supporting Information and Figure S5). Taken together, therefore, these results show that cellulase activity in dynamic multienzyme clusters involves both Cel7A and Cel7B present at the same time and requires the two enzyme types to operate together in close physical proximity.

### Mechanistic Principle of Endo–Exo Synergy Revealed: The Exocellulase Enabled Faster Completion of Its Processive Catalytic Cycle

**Figure 4:**
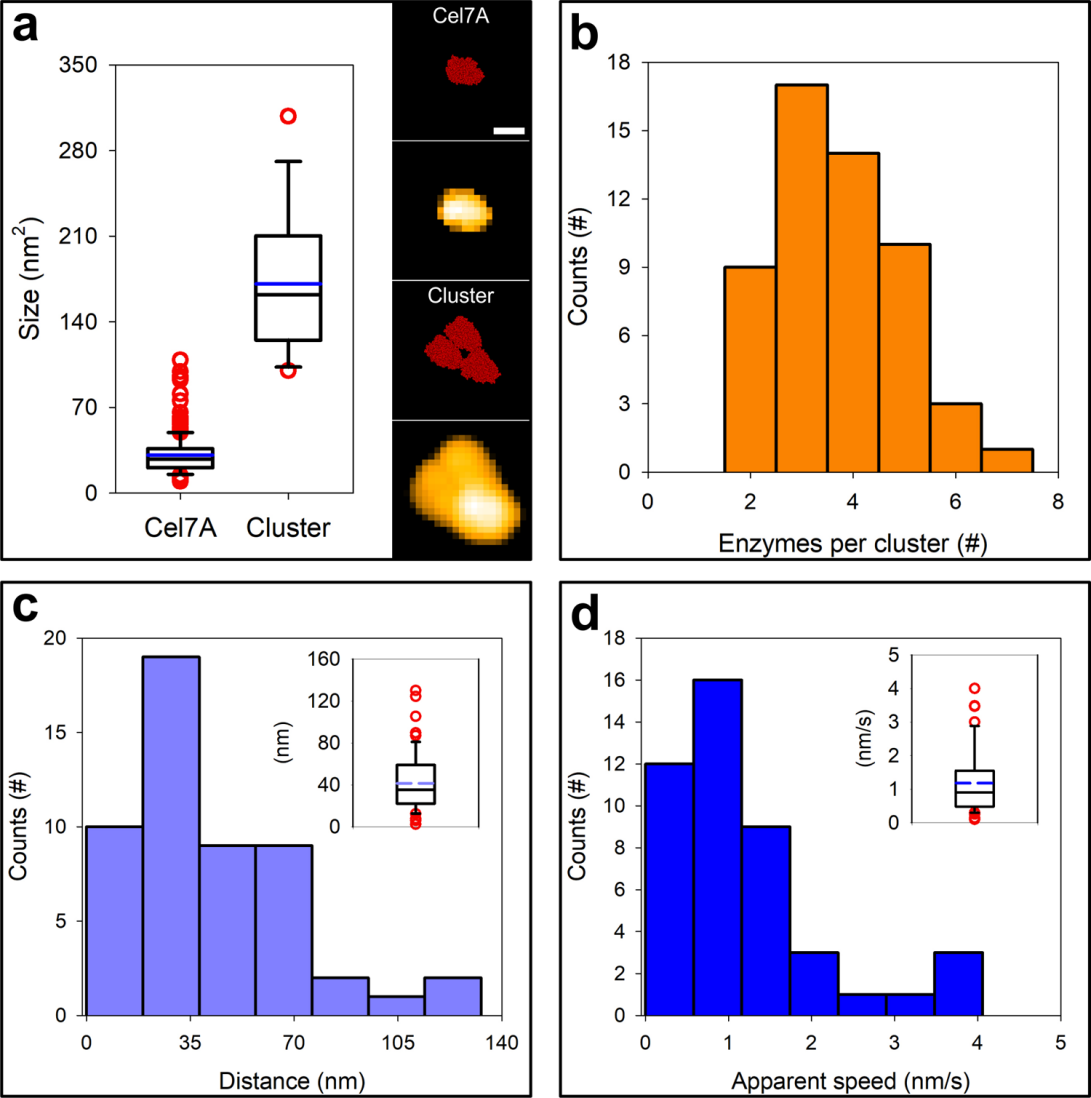
Molecular characteristics of transient clusters of endo- and exocellulase. (a) Comparison of the apparent surface areas occupied by an isolated Cel7A molecule and by cellulase clusters, as analyzed in boxplots of the experimental data (left) and in simulated AFM height images of the enzymes (right). Details regarding the simulation can be found in AFM analysis—Cluster Size, Speed and Degradation in the Supporting Information. The scale bar is 5 nm. (b) Distribution of the number of enzymes found in the cellulase clusters analyzed. (c, d) Distance traveled (c) and apparent speed (d) of enzyme clusters during multilayer-processive degradation of cellulose fibrils. Both datasets were also plotted as a boxplot (shown as insets in the corresponding panels). The mean traveled distance and mean apparent speed were calculated to be 40 nm and 1.2 nm s^–1^, respectively. Medians were calculated to be 35 nm and 0.9 nm s^–1^ for traveled distance and apparent speed, respectively. Boxplots were constructed as follows: the median is indicated by a black line, while the mean is shown in color and boxes extend from the 25th to the 75th percentile of each group’s distribution. Whiskers show the 10th and the 90th percentile, respectively. Outliers are plotted as red dots.

The transient enzyme clusters formed in the reactions of the whole *T. reesei* cellulase and the Cel7A–Cel7B mixture (molar ratio 1.0:1.1) were analyzed in detail (see Preparation of Dispersed Cellulases and Preparation of Isolated Cellulases Cel7A and Cel7B in the Supporting Information). Judged from their projected area, the clusters were about 5-fold larger in size than an individual Cel7A molecule (Figures 4a and S6). From the total set of clusters analyzed (*N* = 93), we determined that the number of enzymes engaged in the cluster formation is centered at 3–4, as shown in Figures 4b, S7, and S8. Each cluster was analyzed for processive movement. The average size and speed of the processive step were 40 ± 28 nm and 1.2 ± 0.9 nm/s, respectively, as shown in Figure 4c,d. Considering the average height (∼3.5 nm) of the fibrils degraded in the processive step, we infer from the literature-derived correlation between the cellulose chain number and the diameter of rod-shaped cellulose fibrils (see Cleavage rate calculation, Figures S9 and S10) that ∼24 cellulose chains are depolymerized in the process. The ∼1 nm length of the chain’s repeating unit cellobiose^46^ implies a cleavage rate of 29 (=24 × 1.2) cellobiose molecules/s. Taking the average cluster to involve 3.5 enzymes (Figure 4b), we estimate the single-enzyme turnover rate to be 8 s^–1^ (Supporting eq 1). This turnover rate is well comparable to the single-step rate of Cel7A for processive cellulose chain cleavage, as obtained experimentally from other single-molecule AFM studies (5.3–7.1 s^–1^)^35,37,47^ or by computational analysis (∼6.9).^48^ However, it is considerably higher than the biochemically determined overall turnover rate (*k*_cat_^app^) of Cel7A, acting alone (∼0.1–0.3 s^–1^)^34,49^ or in the presence of endocellulase (1.5 s^–1^).^10^ The *k*_cat_^app^ of Cel7A in the presence of endocellulase was obtained at a lower temperature (25 °C) than used here (35 °C). Our discussion excludes the possible effects that variable temperature used in the different studies could have had on the enzymatic rate.

**Figure 5:**
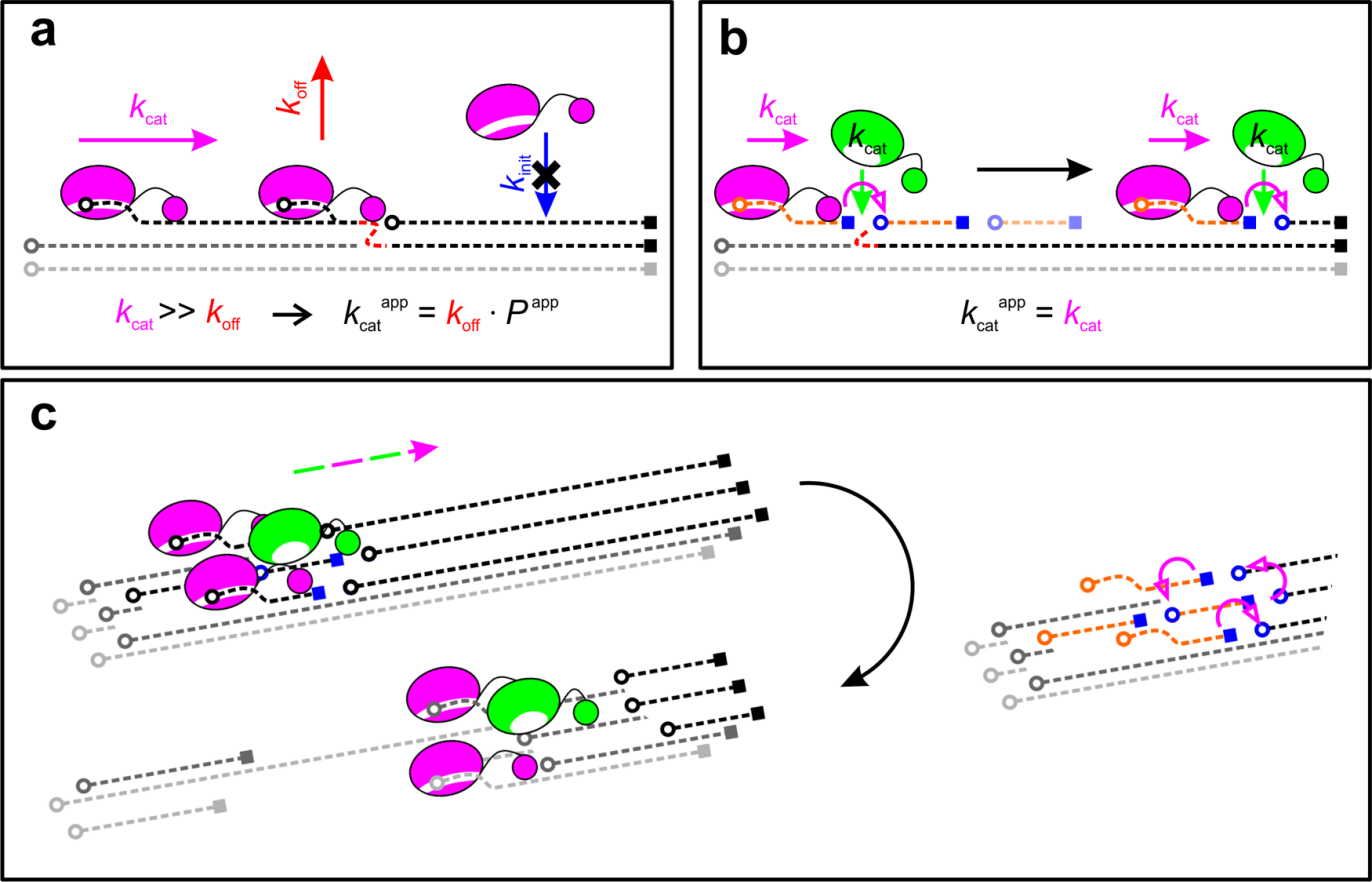
Endo–exo synergy in transient clusters of cellulase and efficient multilayer-processive degradation of cellulose fibrils enabled by it. (a) The turnover rate of the exocellulase observed biochemically (*k*_cat_^app^) is up to 10^3^-fold lower than the rate of the single processive step (*k*_cat_, see ref (10) for details). It is limited by slow enzyme release from the cellulose chain (*k*_off_), processive length (*P*^app^), and additional effects of nonproductive binding, leading to a low apparent rate of initialization of the processive cycle (*k*_init_). Nanoscale obstacles, indicated as red circles, result in enzyme stalling and require a *k*_off_ event for a new processive cycle to start. Note the requirement for slow “de-threading” of the cellulose chain from the exocellulase binding pocket for enzyme release. (b) Endocellulase activity in transient enzyme clusters enables the exocellulase to the realization of its full catalytic potential (*k*_cat_^app^ = ∼*k*_cat_). Short cellulose chains (shown in orange) generated by the endocellulase eliminate the kinetic significance of the *k*_off_ by avoiding the de-threading. They additionally promote the *k*_init_ by creating a high local density of chain ends as productive binding sites (blue circles). Note that the *k*_init_ event is not limited to the original chain but includes all chains locally accessible to the dynamic conformational ensemble of the single exocellulase molecule. For an easier view, the endocellulase is shown as not adsorbed to the cellulose surface. (c) Multilayer-processive degradation of the cellulose fibril. The shown three-enzyme cluster makes a coordinated movement (colored arrows) initiated by endocellulase-catalyzed chain cleavages upstream of the exocellulases in the cluster. Processive degradation by the exocellulases happens on multiple chains in several layers of the cellulose (top layer, black; lower layers in gray shade). The magenta arrows indicate the switch between cellulose chains by the exocellulases. The overall effect is a unidirectional processive degradation of the whole fibril.

The large difference between the single-step and overall turnover rate of Cel7A is explained by the kinetic significance of enzyme dissociation from the cellulose chain (*k*_off_, see Figure 5a). A number of studies^9,11,43,48^ show that the step represented by the *k*_off_ is rate-limiting for the *k*_cat_^app^ and that it is at least 1 order of magnitude slower than the single-step processive rate. The *k*_off_ was determined from single-enzyme tracking AFM as ca. 0.12–0.20 s^–1^.^37,47^ Using biochemical methods, it was determined to have a considerably lower value of just ca. 0.0007–0.01 s^–1^.^4,50^ The *k*_cat_^app^ is obtained with the relationship, *k*_cat_^app^ = *k*_off_·*P*^app^, where *P*^app^ is the apparent processivity (i.e., the number of cellobiose cleavages in a single processive run).

A fundamentally new mechanistic interpretation of the cooperativity between endo- and exocellulases emerges from these results. Activity of the two cellulase types in dynamic multienzyme clusters appears to be spatiotemporally coordinated and mechanistically concerted, as illustrated in Figure 5. It locally concentrates the catalytic interplay between exocellulase (Cel7A) and endocellulase (Cel7B) to benefit the processive hydrolysis of Cel7A intrinsically (Figure 5a,b). Due to the accelerated release of Cel7A from the attacked cellulose chain when the endocellulase cleaves the same chain suitably upstream of the bound Cel7A, the Cel7A can complete its processive cycle ≥ 100-fold faster than when acting alone. Increased dynamics of cellulose chain exchange is evidently crucial for the Cel7A to efficiently attack the three-dimensional array of substrate chain ends presented to it during microfibril degradation by the Cel7A/Cel7B cluster (Figure 5c).

Contrary to when Cel7A is acting on the uppermost cellulose layer of the crystalline surface, unrestricted processivity in the depolymerization of a single cellulose chain no longer determines enzyme hydrolytic efficiency under conditions of activity in a transient cluster with endocellulase. Multiple cellulose chains can be degraded in short processive runs, the length of which appears to be governed by the upstream chain cleavages of the endocellulase (Figure 5b). A second advantage for Cel7A catalysis realizable in dynamic enzyme clusters with an endocellulase is that, due to destabilization of the cellulose chain assembly by endocellulase-promoted internal chain cleavages, the energy requirement for Cel7A to extract a single substrate chain into the enzyme binding pocket can be lowered^4,51^ and the processive chain cleavage thus proceed faster. From computational studies, the intrinsic rate of cleavage of a free cellulose chain by Cel7A is 10.8 s^–1^.^52^ This “optimum” chain depolymerization rate of Cel7A appears to have become largely unmasked in the Cel7A/Cel7B clusters, and it is made available for cellulose chain degradation in a truly efficient, three-dimensional deconstruction process.

## Discussion

### Single-Molecule Dynamics of Endo–Exo Synergy among Dispersed Cellulases

To put the discoveries of the current study into full perspective, it is first instructive to consider the molecular organization of nature’s main enzyme systems for cellulose degradation. Cellulases working as a collective of individual hydrolases (“dispersed” cellulases) are widespread and prototypically exemplified by the *T. reesei* enzymes used here.^4,30,53^ Additionally, there exist specialized systems of so-called “complexed” cellulases.^3,30^ These can be found as multimodular fusion proteins composed of different enzymatic subunits^54,55^ but are most characteristically represented by the cellulosome.^3,56^ The cellulosome is a large multienzyme nanomachine of cellulose degradation, exhibiting nine or more cellulase subunits assembled on a flexible scaffold protein.^3,57^ Contrary to dispersed cellulases, the cellulosome places restraint on the spatial dispersion of its individual enzymes on the cellulose surface. Additionally, it restricts the laterally directed activity of its processive enzyme subunits. The cellulosome thus directs the material deconstruction transverse to the longitudinal axis of the cellulose fiber.^30^ Release of the confining force in dispersed cellulases causes transversal-to-lateral change of the directionality of fiber deconstruction.^30^ The molecular assembly state of cellulases thus determines the nanoscale characteristics of the enzymatic cellulose deconstruction, observable as distinct “fibril cutting” and “surface ablation” modes of substrate degradation by the cellulosome and the dispersed cellulases, respectively.^30^

In a dynamic single-molecule view of the mechanism, synergy between endo- and exocellulases relies on a productive cycle between concentrating the cooperatively acting enzymes locally on the cellulose surface and dispersing them again to enable access to fresh “chain attack” sites.^10,30^ Besides desorption and re-adsorption, dispersion involves molecular diffusion as well as directed movement of the cellulases on the solid surface. From their molecular assembly state, therefore, complexed and dispersed cellulases are biased toward supporting primarily one of the opposed elements (concentration vs dispersion) of the complete synergistic cycle. In placing enzymes of synergetic function in close spatial proximity, the cellulosome maximizes the effect of local concentration. Conversely, dispersed cellulases facilitate a distributed ensemble of individual enzymes on the cellulose surface.

The current study suggests how dispersed cellulases can generate molecular proximity for optimum cooperative function between their endo- and exoenzymes (Figure 5b,c). The cellulases adopt the “enzyme assembly” principle of the cellulosome in a highly dynamic form and are finely adjusted to local features of the substrate nanostructure. By engaging in transient enzyme clusters at specific, endo- and exoenzyme-accessible attack sites on the cellulose, the dispersed cellulases overcome the lack of locally focused usage of endo–exo synergy (Figure 5b,c). The enzyme cluster formation appears to involve biological recognition on the part of the cellulases for nanodomains of the cellulose microfibril that show molecular defects in the polysaccharide chain organization. Assumption of (specific) intermolecular interactions of the clustered cellulases is not required from the results shown, but, of course, it remains an interesting possibility. Dynamic clustering of cellulases can offer a distinct advantage compared to stable complexation. It facilitates spatiotemporal coordination of the interplay of the different enzyme activities. Thus, it can help to maintain endo–exo synergy over the longer course of substrate degradation. Realizing the limits of the classical (“substrate preparation”) interpretation of endo–exo synergy among cellulases, some biochemists have speculated about a possible role of enzyme–enzyme interactions at the cellulose surface.^10,25,58^ However, as Väljamäe et al. noted at the time: “... using known experimental systems, it is impossible to corroborate directly the existence of these loose in situ complexes.”^25^

Our AFM results obtained at high temporal resolution go beyond the important first-time demonstration of transient enzyme complexes in dispersed cellulases. They reveal a mechanistic correlation between the dynamic clustering of endo- and exocellulases, and the nanoscale characteristics of cellulose deconstruction by the enzymes. Since the endocellulases add a distinct transversal component to the primarily laterally directed cellulose deconstruction by the exocellulases, endo–exo enzyme activity in transient clusters of cellulase gives rise to an unprecedented, and apparently highly efficient, multilayer-processive mode of substrate degradation (Figure 5c). Each multilayer-processive step involves enzyme cluster dynamics in three characteristic phases: step-initiating cluster assembly from typically three to four enzymes of mixed endo- and exo-type; joint lateral movement of the cluster-associated enzymes as the fibril deconstruction proceeds; and step-terminating cluster disengagement. The newly discovered mechanism is effective: it contributes up to half of the total cellulose fiber deconstruction by the dispersed cellulases.

### Transient Clusters vs “Traffic Jams” of Cellulases

Igarashi and colleagues^35−37,47^ have shown that Cel7A (and related enzymes from its exocellulase class) can be observed with high-speed AFM to slide unidirectionally along the crystalline cellulose surface. At certain points, several enzyme molecules previously seen to move continuously in the same direction were found to exhibit collective halting, as in a molecular traffic jam caused by an obstacle. In AFM images, the jammed Cel7A molecules appear as clusters of multiple enzymes,^35^ just as the ones observed in the current study. However, the two types of enzyme cluster differ fundamentally in the dynamics of their formation as well as in their functional role in cellulose degradation. The jammed Cel7A clusters result from several enzyme molecules running unidirectionally into an obstacle.^35^ The clusters of endo- and exocellulase form primarily due to localized enzyme binding (Figures 2b,c and 3a, Movies S2 and S4). Once the obstacle is removed (e.g., due to the “pushing force” of multiple queued enzymes as proposed by the authors), the Cel7A clusters dissolve by single enzyme molecules starting to move again individually.^35^ Clusters of endo- and exocellulase however exhibit joint movement of the enzymes involved (Figures 2b,c and 3a, Movies S2 and S4). Cel7A traffic jams halt the progress of cellulose chain depolymerization.^35^ In spite of the fact that several Cel7A molecules get trapped in a transient cluster, the substrate degradation is still restricted to the immediate surface, or the outer shell, of the cellulose substrate.^35^ In contrast, the clusters of endo- and exocellulase observed here exhibit coordinated movement, three-dimensional degradation, and operate close to their full catalytic potential while lacking the arresting feature of traffic jams.

### Enabling Processive Turnover to Full Speed in Multilayer Cellulose Deconstruction

In a widely held view of the exocellulase mechanism, there exists a trade-off between the processive length (the number of cellobiose units released in the processive run) and the turnover rate for the complete processive cycle.^37,43,59^ Under reaction conditions not limited by the substrate and in the absence of other enzymes generating a synergistic effect, the turnover rate for the single exocellulase (Cel7A, but also various other enzymes from the same class) appears to be controlled by dissociation from the substrate.^9,10,43,48,60^ A plausible structural interpretation is offered by the requirement of dissociation to release the oligosaccharide chain threaded into the narrow substrate binding tunnel of the enzyme.^4,52,61,62^ The *T. reesei* Cel7A exhibits a high processive length (*P*^app^ = 61 ± 14;^9^ 88 ± 10^63^) on bacterial cellulose, and its processive turnover rate *k*_cat_ was determined from single-molecule high-speed AFM studies as ∼7.1 s^–1^.^35^ The considerably lower *k*_cat_^app^ determined biochemically (ca. 0.1–0.3 s^–1^)^34^ includes effects of nonproductive binding of the Cel7A on the cellulose surface.^62,64^ Besides enzyme blocked in the “*k*_off_ state”, Cel7A adsorption at sites of the cellulose that fail to initiate a processive cycle appears to be relevant in particular. AFM evidences indeed show only a fraction of the adsorbed Cel7A molecules to engage in continuous directional movement associated with processive chain depolymerization.^35,37,65^ Protein engineering of Cel7A for improved activity in cellulose hydrolysis has often targeted the substrate tunnel with the aim of speeding up the dissociation.^34,62,66−69^

Working individually at the surface of crystalline cellulose, an exocellulase evidently benefits from exhibiting high processivity.^4,37,70^ The transition between processive cycles arguably involves “resting periods” of nonproductive binding, resulting when the (partly stochastic) physical processes of enzyme molecular dispersion on the cellulose surface (i.e., desorption/re-adsorption, on-surface diffusion) fail in positioning the enzyme suitably for activity. Of note, each processive cycle requires the extraction and initial threading of a single cellulose chain from the solid material.^4,48,62^ The ≥ 100-fold difference in single-molecule compared to ensemble-averaged turnover rate of Cel7A (*k*_cat_^app^) might plausibly originate from nonproductive binding restricting the portion of “catalytically engaged” enzyme in total cellulase adsorbed on the cellulose. To be sure, our discussion recognizes stalling of the moving enzyme due to molecular/nanoscale “obstacles” encountered on the surface (*k*_off_). However, unlike stalling that has received much interest mechanistically^10,26,64,71−73^ and in regard to engineering better cellulases,^66,74−76^ nonproductive binding as “standby adsorption” appears to have been overlooked as a molecular factor of the enzymatic degradation rate. On a crystalline cellulose surface, a low frequency of productive encounter with accessible substrate chains may limit the enzyme activity considerably more than running into an obstacle.^50,72,77,78^

However, microfibril nanodomains of low order in the cellulose chain organization can severely restrain the activity of processive exocellulases due to overlapped effects of stalling and nonproductive adsorption (Figure 5a). Dispersed cellulases exploit transient assembly into enzyme clusters to direct the cooperative activity of their endo- and exoenzymes toward the complete elimination of these rate-retarding factors of cellulose chain depolymerization by the exocellulase, as depicted in Figure 5. The spatiotemporally coordinated activity of endo- and exoenzymes present in close proximity enables the exocellulase to full usage of its processive speed in multichain cellulose degradation. Short cellulose chains generated by endo cleavage and partly detached from the solid material can be degraded fully by the exocellulase. This effectively shuts out the slow dissociation step from the exocellulase processive cycle. Due to the enhanced local density of accessible chain ends brought about by endolytic activity, the productive binding of substrate by the exocellulase is strongly facilitated. Simulation study of endo–exo synergy by single-molecule stochastic modeling suggested enhancement of the “exo complexation rate” (= overall rate of recruitment and threading of the substrate chain by the exoenzyme) by endoenzyme activity as a fundamental requirement for cooperativity between the two types of cellulase.^10,12^ Our results yield a mechanistic interpretation of that enhancement in terms of a local concentration effect on the substrate chains made available to the exocellulase. The simulation study further predicted that increased surface roughness generated by the endo activity would result in enhanced stalling of the exocellulase.^12^ We show here that endo–exo activity in transient enzyme clusters not only overcomes the possibility of such kind of “negative synergy”, but effectively turns it into an advantage, to enable a truly three-dimensional, multilayer-processive deconstruction of the cellulose microfibril (Figure 5). Results of the current study might also be useful to revisit mechanistic interpretation of the curious phenomenon of cellulase inhibition by the cellulose substrate.^78,79^ Studying binary mixtures of endo- and exocellulases on bacterial cellulose, Väljamäe et al.^78^ found substrate inhibition dependent on substrate concentration, substrate pretreatment (e.g., to increase the number of free chain ends; to remove amorphous substrate parts) and enzyme concentration. The substrate inhibition was originally interpreted in terms of the classical view of endo–exo synergy.^78^ The relative contribution to the overall sugar release rate resulting from the activity of enzymes in transient clusters may change (e.g., decrease at high substrate concentration) depending on the variation of different reaction parameters. Overall, the proposed mechanism of endo–exo synergy among cellulases presents a new paradigm of efficient interfacial catalysis by enzymes in the degradation of structurally organized polysaccharide biomaterials.

## Supporting Information Available

Detailed description of the materials and methods involved in the preparation, purification, and characterization of bacterial cellulose and fungal cellulases; sample preparation for AFM observations; AFM operation, calculations, AFM image processing, and data analysis; multilayer degradation of an isolated fibril at reduced enzyme loading (Figure S1); analysis of cellulose fiber degradation by Cel7A (Figure S2); isolated Cel7A at high concentration deconstructing a cellulose fiber (Figure S3); degradation of cellulose using isolated Cel7B (Figure S4); degradation of cellulose pretreated with Cel7B by Cel7A (Figure S5); analysis of the user-induced bias in cluster area measurements (Figure S6); in silico AFM measurements of different cluster configurations (Figure S7); analysis of the user-induced bias in counting the number of enzymes in the clusters (Figure S8); calculated number of cellulose chains that fit into rod-shaped cellulose fibrils with different diameters (Figure S9); dimensional properties of a simulated cellulose fibril (Figure S10); and flowchart summarizing the steps of the image processing procedure performed by the in-house developed MATLAB routine (Figure S11) (PDF)
Dispersed cellulases from *T. reesei* employing two different modes of action during cellulose deconstruction (Movie S1) (AVI)
Formation of transient clusters of dispersed cellulase during degradation of bacterial cellulose microfibrils (Movie S2) (AVI)
Overloading of Cel7A does not result in enzyme clustering behavior (Movie S3) (AVI)
Degradation of cellulose fibrils by Cel7A in the absence and presence of Cel7B (Movie S4) (AVI)

## Supplementary Material

cs2c02377_si_001.pdf

cs2c02377_si_002.mov

cs2c02377_si_003.mov

cs2c02377_si_004.mov

cs2c02377_si_005.mov

## Abbreviations

AFM: atomic force microscopy
HOPG: highly ordered pyrolytic graphite
GP: gradient parameter
MP: median parameter
