# Supplementary material for: Enzyme Synergy in Transient Clusters of Endo- and Exocellulase Enables a Multilayer Mode of Processive Depolymerization of Cellulose
Source: ACS Catal. 2022 Aug 24;12(17):10984–94. doi: 10.1021/acscatal.2c02377 (PMC9442579; doi:10.1021/acscatal.2c02377)
Supplement: Supplementary file 1 — cs2c02377_si_001.pdf [file cs2c02377_si_001.pdf]

Supporting information

Enzyme Synergy in Transient Clusters of Endo- and Exocellulase Enables a Multilayer Mode of Processive Depolymerization of Cellulose

*Krisztina Zajki-Zechmeister[1], Manuel Eibinger[1], Bernd Nidetzky[1,2]**

[1]Institute of Biotechnology and Biochemical Engineering, Graz University of Technology, Petersgasse 10-12/1, 8010 Graz, Austria.

[2]Austrian Centre of Industrial Biotechnology, Petersgasse 14, 8010 Graz, Austria.

Corresponding Author

*Correspondence should be addressed to B.N. (bernd.nidetzky@tugraz.at)

The supplementary information file includes:

Detailed description of methods

Supplementary Figures S1 to S11

Captions for Supplementary Movies S1 to S4

Supplementary References

Methods

Unless stated, all chemicals were of the highest purity available from Carl Roth + Co KG (Karlsruhe, Germany).

Preparation of single bacterial cellulose fibers

Acetobacter xylinum (46602, DSMZ-German Collection of Microorganisms and Cell Cultures GmbH) was cultivated in sterile yeast peptone mannitol medium (360, DZM) as described comprehensively in our earlier work (1). The main culture (200 mL) was inoculated with 2 mL of the pre-culture and incubated in a static condition at 30 °C for 14 days. This resulted in two cellulose films. The first film was formed during the first 7 days and was removed. The second one, which was formed during the remaining 7 days, was collected and cleaned for further usage. Cleaning included rinsing with ultrapure water and incubation in 1 M sodium hydroxide over night at 4 °C and mild magnetic stirring. Residual cell debris, media components and sodium hydroxide were removed by repeated washing and incubating in ultrapure water until the water bath reached a pH of 7.0. To extract isolated fibers from the film, firstly it was disentangled with a conventional hand blender in ultrapure water. Heavy dilution of the suspension (< 0.01 g/L) prevented hydrogel formation. The mechanical disintegration was continued with ultrasonic homogenization performed with a Sonic Dismembrator (Fisherbrand Model 505, Fisher Scientific GmbH,) operated with a 6A/4V titanium alloy probe head (Fisherbrand™ FB4406, Fisher Scientific GmbH) in pulse mode: A 2 s pulse (40% intensity) was followed by 5 s pause for a total pulse-time of 20 min. The suspension was cooled in an ice bath for the duration of the whole process. This method resulted in a bacterial cellulose suspension with single fibers gently released from the three-dimensional network of the film. It was stored at 4 °C.

Preparation of dispersed cellulases

A complete cellulase mixture was obtained from *Trichoderma reesei* (Strain SVG17, Institute of Biotechnology and Biochemical Engineering, Graz University of Technology), which is a well-known

producer of cellases (2). *T. reesei* was grown on wheat straw and harvested as described in our previous study.(3) In brief, the supernatant was clarified via centrifugation (4420 g at 4 °C) for 20 min through a glass microfiber filter (Whatman). The purified cellulase mixture was supplemented with 0.05% w/v sodium azide and stored at 4 °C. SDS PAGE analysis (data not shown) confirmed the cellulase composition known from literature(4).

Preparation of isolated cellulases Cel7A and Cel7B

The major fungal exocellulase Cel7A was isolated from the *T. reesei* supernatant, as described earlier.(5) In brief, the buffer of the complete system was exchanged to 20 mM triethanolamine with pH 7.0 using centrifugal concentrators (10 kDa, Vivaspin Turbo, Sartorius). Purification was executed using an ÄKTA FPLC (Amersham Biosciences) where the mixture was loaded onto a 6 mL pre-packed column (Resource Q, GE Healthcare) equilibrated with the same buffer. The purification was performed with a linear gradient of 0 – 300 mM sodium chloride over 10 column volumes. Only the desired Cel7A elutes at about 180 mM sodium chloride, which facilitates precise collection of the enzyme. The buffer of the isolated Cel7A was exchanged to 50 mM sodium acetate with pH 5.0 and it was stored at 4 °C. SDS PAGE analysis (data not shown) showed a single protein band approaching 65 kDa in accordance with the molecular size of the enzyme.

The major endocellulase Cel7B was commercially obtained from Megazyme. Note, that Cel7B originates from *Trichoderma longibrachiatum*, which is an anamorph of *T. reesei*. It is almost identical to its counterpart from *T. reesei* regarding their amino acid sequence (identity: 95%, similarity: > 95%). Cel7B was stored as provided at 4 °C. Prior to experimental use the buffer was exchanged to 50 mM sodium acetate buffer, pH 5.0, using disposable centrifugal concentrators with 100 kDa mass cut-off filters (Vivaspin Turbo 15, Sartorius).

Protein concentration of the complete cellulase mixture was measured using a commercially available kit (Pierce BCA Protein Assay Kit, Thermo scientific). Concentrations of the isolated enzymes were measured with UV absorbance at a wavelength of 280 nm, using their respective molar extinction coefficients of 86,760 $M^{-1}cm^{-1}$ for Cel7A and 74,940 $M^{-1}cm^{-1}$ for Cel7B. (1)

Atomic force microscopy – In situ degradation experiments

In situ observation of cellulose degradation by the cellulases was done with a commercially available atomic force microscope (AFM, Dimension Fast Scan Bio, Bruker) equipped with a commercially available controller (Nanoscope V, Bruker) and software (Nanoscope 9.2, Bruker). Measurements were performed in liquid environment using tapping mode with FastScan DSS probes (Bruker) as described in our resent work. (1) The liquid environment was heated with a temperature controller (Bruker) to 35 °C. Single cellulose fibers were immobilized on highly oriented pyrolytic graphite crystals (HOPG, Grade I, SPI supplies) by incubating the freshly cleaved surface with 300 µL of single cellulose fiber suspension for 15 min. The surface was rinsed with deionized water. Residual droplets were removed by spraying carbon dioxide for 2-3 s. The HOPG crystal was mounted onto the AFM stage. To create the liquid environment, 250 µL of sodium acetate buffer was pipetted on the crystals surface. The scan head was gently immersed into the buffer drop. In the heated liquid environment, the probe needed to equilibrate for 10 min prior to measurement to ensure good detector alignment and stable measurement conditions. Engagement was performed automatically using the "smart engage" setting. After a stable contact between tip and surface was ensured, 30 µm × 30 µm areas were systematically scanned for isolated, firmly attached fibers. As soon as a suitable fiber was detected, 10-100 µL of the relevant enzyme preparations were carefully injected in 10 µL portions. Experiments with the complete cellulase system from *T. reesei* (15 µg/mL) were loaded with 10, 20 and 100 µL. Experiments with the Cel7A/Cel7B mixture as well as isolated Cel7A and isolated Cel7B were performed by adding 20 µL Cel7A (15 µg/ mL) and/or 20 µL Cel7B (15 µg/mL), respectively. The experiments using Cel7A at elevated protein concentration were performed by adding 30 µL and 100 µL of a more concentrated stock (2000 µg/mL).

The deconstruction was observed at least for 2 h or until the fiber was completely gone. During long measurement times (> 3 h) it was necessary to inject additional buffer due to evaporation.

Atomic force microscopy – Device parameters during measurement

As described in full detail in our previous work (1), AFM parameters were selected and adapted with cautious sensitivity based on the feedback from the device. Summarily, topography, phase and

amplitude images were measured for every frame. The Drive Amplitude was adjusted by observing the quality of the phase image, the Amplitude Setpoint was regulated to just below contact (~70-90% of free amplitude). Usually, the same Setpoint was used during a measurement, however, in case of any drift of the z-piezo, it was adjusted accordingly. The Integral Gain was continuously set just below resonance, the Proportional Gain was kept at a value about 3-5 times of the Integral Gain value. The Z-range of the scanner was decreased by half its maximum value.

In contrast to our earlier work, the main objective was to illuminate enzyme behavior during degradation rather than degradation patterns visible on the cellulose substrate. For this, higher time resolution was necessary. Switching from globally observed fibers to locally acting enzymes allowed for smaller scan areas (0.0500 μm^2 < scan area < 0.0025 μm^2), hence faster measurements were possible. The scan rate was increased until the image quality could not be improved any further just before the point of obscuring the enzymes due to measurement artefacts. To exhaust the maximal possible scan speed, the amount of measurement points was reduced by choosing larger pixel step sizes. This method enabled measurement speeds of up to 2 frames/s.

Application of Cel7A as single enzyme at different concentrations.

Isolated Cel7A was investigated with respect to cluster formation. Experiments were performed by adding 20 µL of Cel7A at a concentration of either 15 µg/mL or 2000 µg/mL to our standard AFM setup (see *Atomic force microscopy – In situ degradation experiments*). At the lower concentration, Cel7A was observed on the cellulose surface continuously adsorbing on fibril defect locations or endings (Fig. **S2a**). Degradation occurred along the longitudinal axis of the fibrils, resulting in continuous ablation (Fig. **S2b**). Interestingly, the constant fiber loss decelerated after 40 min and 8 % deconstruction. No cluster was observed throughout the measurement.

The use of Cel7A at the higher concertation led to a rapid disappearance of the fiber (after ~34 min), however, the deconstruction process was clearly different from that of the complete cellulase system as well as the Cel7A/Cel7B mixture (Fig. **S3**, Movie **S3**). A large number of

Cel7A molecules were observed to absorb, aggregate and slide longitudinally along the fiber surface. The deconstructions occurred in an ablation style, while discrete steps of multilayered fibril removal were absent. Note that a major part of the fiber was destabilized and removed by the AFM tip.

Preparation of Cel7B-treated cellulose fibers and their subsequent degradation by Cel7A.

Bacterial cellulose fibers were pretreated with Cel7B as follows. Five mL of a single fiber suspension (< 0.1 mg/L) were adjusted to pH 5.0 by adding 1 mL of 300 mM sodium acetate buffer (pH 5.0). Subsequently, 1 mL of this suspension was transferred to a Thermomixer comfort (Eppendorf) and heated to 35 °C, which corresponded to the conditions in the AFM experiments. 20 µL Cel7B (15 µg/mL) were added to the reaction volume. After 1 h, the reaction was stopped by adding 100 µL of 1 M sodium hydroxide. The suspension was stored at 4 °C and used within 24 hours. The pretreated fibers were used as substrate for Cel7A in the AFM measurement, which was performed similarly as described in *Atomic force microscopy – In situ degradation experiments*. The effect of Cel7A was observed continuously on the cellulose surface (Fig. **S5a**), and after 2 h, 4 % of the total fiber volume was removed by ablation and thinning (Fig. **S5b**). Interestingly, the deconstruction decelerated after 50 min. The same behavior was observed for Cel7A acing on untreated cellulose fibers. No formation of clusters was observed.

AFM analysis – Cluster size, speed and degradation

The size of the clusters was assessed with common tools in Gwyddion (Version 2.60). (6) In more detail, the cluster was manually masked by an operator and the projected area of said mask was determined with the "measure individual grains" tool in nm^2. To account for uncertainties regarding the exact edges of the flexible clusters, two different operators independently assigned masks to the same clusters, resulting in an average difference of ~20%, being ~50 nm^2 (Fig **S6a**, **b**). This procedure

was also performed for the isolated exocellulase Cel7A to obtain a reference value for single enzymes (Fig. **4a**).

However, enzymes within clusters do not necessarily assemble in a linear fashion on a plane surface but rather on a curved surface and, thus, the projected area of a cluster does not scale linearly with the enzyme amount. Consequentially, the number of enzymes involved in a cluster cannot readily be accessed by dividing the projected cluster area by the projected area of a single enzyme. Therefore, different configurations of enzymes on a cellulose fibril were measured in silico with the BioAFMViewer (7) and manually compared to the actually measured AFM height images by two independent operators for a more accurate estimation.

For this it was necessary to generate PDB files resembling different enzyme scenarios, e.g., one Cel7A and one Cel7B on the edge of a microfibril (Fig. **S7a**, 1:1). The files were generated using Pymol (Version 4.6.0). Structure information for Cel7A and Cel7B were taken from the Protein Data Bank website (https://www.rcsb.org/) with file IDs 4d5q and 1eg1, respectively. Note, that these structures only resemble the catalytic core of the enzymes, without linker or binding module and therefore will appear smaller in the in-silico measurement compared to the actual AFM measurement. Structure information for the microfibril was generated using the cellulose builder. (8) Settings were chosen as follows: Phase: I-ALPHA, PBC: none, PCB_c=false, select input: fibril, input 1:20). This resulted in a microfibril with the often reported 36 chain structure having a length of 20 cellobiose units. Using Pymol, both enzyme types were arranged on the microfibril surface as well as on the edge. The chosen Cel7A/Cel7B mixtures were as follows: 1 Cel7A + 0 Cel7B, 1 Cel7A + 1Cel7B, 2 Cel7A + 1 Cel7B and 3 Cel7B (Fig. **S7a**). Note, that the arrangement in Pymol happened only considering the geometry of the components and therefore disregarding any physical or chemical surface interactions. However, this does not limit our use case, as we only employ these created scenarios as visual aid for the estimation of enzyme number. These scenarios were exported as PDB files and put into the BioAFMViewer for in silico measurements. Settings were chosen to mimic the geometry of the tip used in the experiments (FastScan DSS): Scan step: 1 nm, cone angle: 15. The tip radius was varied between 1 nm (Fig. **S7b**), 2 nm (Fig. **S7c**) and 3 nm (Fig. **S7d**) to prevent over-counting caused by a

broad tip. The scan area was set to 150 x 100 nm. Cutting the actually measured cluster frames to the same dimensions enabled a side-by-side comparison between the appearance of the in situ and in silico measured clusters. Again, with this comparison method, two different operators evaluated the enzyme count within the clusters, which led to an average difference of 0.7 enzymes (Fig. **S8**).

Analysis regarding the volumetric degradation behavior was performed as described previously. (1) Briefly, for every AFM frame within one sequence, the sum of all height pixel entries was calculated ("fiber volume"). As the fiber was continuously deconstructed, also the overall sum of height pixel decreased. The first fiber volume was defined as 100% and by successively subtracting the fiber volume of the consecutively following frames the loss of fiber height can be tracked throughout the measurement.

Calculation of the processive turnover rate of cellulase clusters

To calculate the efficiency of the multienzyme clusters as a whole, the number of cellulose chains within the degraded fibril had to be estimated. An often used and widely accepted geometrical representation of bacterial cellulose microfibrils is that of a ribbon with a rectangular cross-section (9,10). However, the reported dimensions and aspect ratios of the cross-sectional area of these ribbon-shaped fibrils vary by a factor of up to 4 between different studies (9,11). Hence, in this study, the fibril was assumed to have a cylindrical shape with a consequently circular cross section. Assuming that the cross-section of cellobiose is ~0.4 nm^2 (see ref. (12) for details), it was calculated how many cellobiose units could theoretically fit into fibrils with various diameters (Fig. **S9**, adapted from ref. (13)). The fibril diameters in our fibrils were measured directly as the fibril height in the AFM experiments. Fibril heights ranged from 2 to 4 nm (Fig. **S9**, highlighted in grey) representing about 8 to 36 cellulose chains. On average, the fibril height was 3.5 nm, hence containing 24 chains (Fig. **S9**, green x symbol). This estimate was further validated by measuring the 36 chain model of the fibril in silico using the BioAFMViewer. (7) The cellulose file was generated using the cellulose builder (8) as described above. The model of the fibril (Fig. **S10a**) was measured with the AFMBioViewer with settings resembling the actual experiments (scan step: 1 nm, cone angle: 15 °, tip radius: 2 nm) (Fig. **S10b**). Analysis of the horizontal and vertical profiles of the simulated fibril indicated a theoretical

height of 4 nm for 36 chains (Fig. **S10c**). Regarding the experimentally observed height of ~3.5 nm, our estimation with 24 chains was taken as prototypical fibril.

Combining the assumed number of chains, n_{CB}, in one microfibril, the reported length of a cellobiose unit (12), l_{CB}, the average cluster velocity, v_C, and the average number of present enzymes, n_C, led to calculation of the turnover rate (k_{cat}).

$$k_{cat} = \left(\frac{n_{CB} \, * \, v_C}{l_{CB}}\right) * n_C^{-1} \tag{1}$$

AFM image processing and video construction

AFM images shown in the time-lapse movies and used for pixel based calculations were carefully preprocessed with a dedicated and automated MATLAB routine (developed in R2017b, Version 9.3.0.713579, Fig. **S11**), described in full detail in our recent work (1). Especially for the image sequences used for quantitative evaluation it was important to correct common measurement artifacts in a meaningful way, without introducing new correction artifacts or obscuring the physically measured features. This was achieved by designing the routine according to the usually employed successive correction steps performed in established software, e.g., Gwyddion. (6) Overall, it involves 5 main steps: object masking, data manipulation, scaling, drift correction and analysis. All topography data was processed in this manner, whereas any phase and amplitude channels were only processed regarding scaling and drift correction.

Identification and separation of objects (cellulose fibers and enzymes) and background (HOPG) were done by identifying edges and surfaces with user set gradient and median parameters. Both user defined parameters were set regarding only the first image of a sequence. Correct settings of the parameters were fundamental for a high-quality processing of the data. Therefore, they were optimized until >90% of the pixels were accurately assigned either to object or background. Once suitable parameters were found, they were not changed within the sequence, resulting in individual masks for every frame within one sequence calculated with the same settings.

Assigning every image frame with its mask allowed for automated data manipulation to correct the raw data from measurement artifacts in an efficient way. This was especially important for measurement sequences consisting of over hundreds of frames. Furthermore, time dependent analysis, regarding the change of the object (in our case cellulose deconstruction), was made possible.

The first manipulation step corrected for tilts in the images. This was done by calculating a plane that fitted best to the 3D data points given by all pixels defined as background in the masking step. The distances (perpendicular to the plane) between all points and the plane were minimized using least square method. The resulting plane was subtracted from the whole image (background + object pixels), thereby correcting the tilt.

The second step corrected for mismatched baselines of rows in fast scan direction, by fitting a polynomial of degree 1 to the background pixels with least square method. The fitted lines were successively subtracted from all pixels in the individual rows. Rows consisting of > 80% object pixels were not corrected as the small amount of background pixels often were not enough input for successful fitting but would rather introduce calculation artifacts.

Consistent false-color scaling was performed by setting the lowest value for every image to zero. Once all frames were set to zero, one user chosen maximum value was defined. All images were scaled to this one maximum value.

Corrected and scaled data sets were exported in original pixel size in portable network graphics (.png, 24-bit depth) for movie generation as well as to 2D matrices containing the, now corrected, actual height information for further analysis.

Phase and amplitude data were not manipulated and solely processed regarding the false-color scale. For the phase channel, the lowest value was set to zero and, similar to the topography channel, one maximum value was chosen to scale all the images to. For the amplitude channel, this routine alone was not sufficient due to controller feedback errors and an asymmetric distribution of amplitude values. Both problems cause affected images to be scaled in the upper false-color region, rendering interesting features hardly visible for the human eye. This was counteracted by defining the minimum

value of the image newly by mirroring the range between maximum and mean value of all pixels, as described in our earlier work. (1)

The last step of preprocessing was drift correction of the image sequence. It was performed similarly for all the channels also in MATLAB as an adaptation from Sugar et al (14). In brief, a routine was created, were a user-chosen reference image is compared to all other images in the sequence. For every image it is determined how many pixels in x- and y- directions it has to be shifted to best match the reference image. To find the best match, and the number of steps to it, the cross-correlation function between both images was calculated.

Supplementary movies were created with Davinci Resolve (Version 17.2.1, Blackmagic Design).

Supplementary Figures

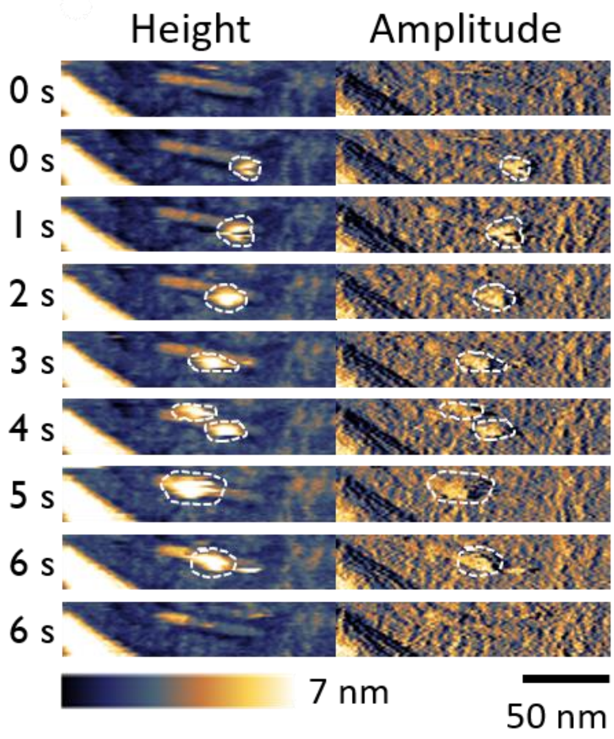

Figure S1. Multilayer degradation of an isolated fibril at reduced enzyme loading. AFM height images, showing multilayer degradation of an isolated fibril through a continuous series of processive steps by an enzyme cluster (enveloped in white). The enzyme loading was reduced by a factor of 5 (15 µL instead of 100 µL at 20 µg/mL). Scale bar and false color scale are included in the figure.

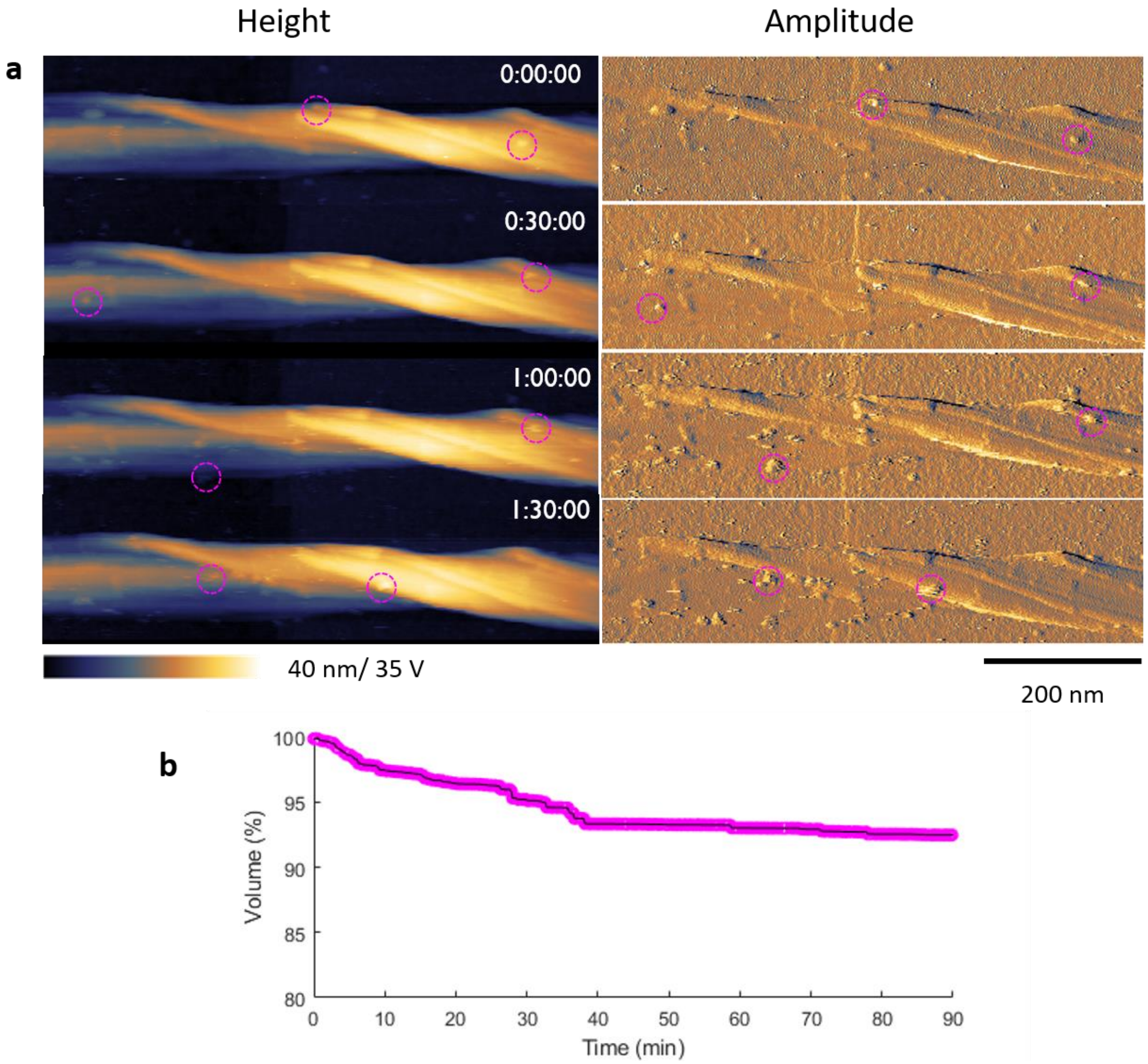

Figure S2. Analysis of cellulose fiber degradation by Cel7A. (**a)** AFM height (left) and amplitude (right) images of fiber deconstruction by Cel7A at reduced enzyme loading (20 µL at 15 µg/mL). Individual Cel7A molecules (magenta circles) can be seen on the fiber surface. (**b)** Tracking fiber volume loss over time showed a biphasic behavior with respect to the rate of degradation. Approximately 8 % of the fiber is removed within the first 40 min, but minimal degradation is observed thereafter. Scale bar and false color scale are included in the figure.

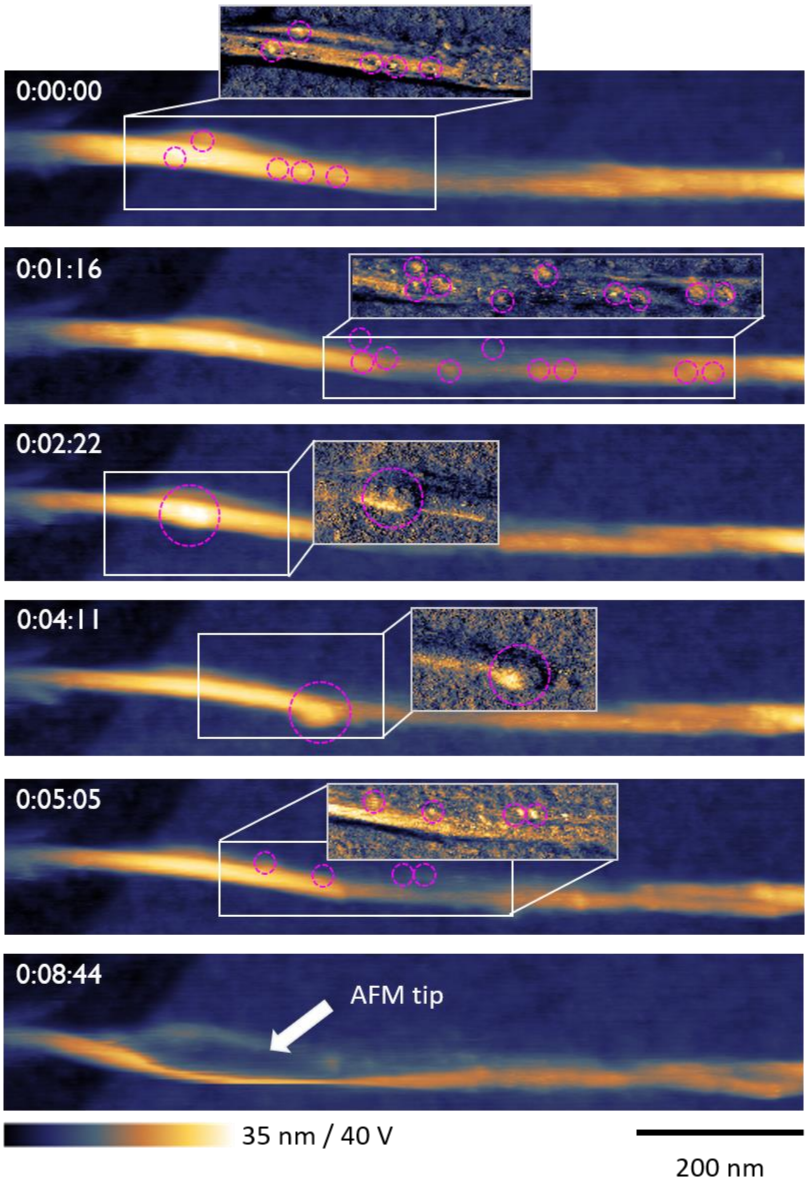

Figure S3. Isolated Cel7A at high concentration deconstructing a cellulose fiber. AFM height images of fiber deconstruction by Cel7A at elevated enzyme loading (100 µL at 2000 µg/mL). Individual Cel7A molecules are highlighted (magenta circles) in the height and amplitude channel (inset). Note that at several instances (see 02:22 and 04:11 min mark) larger and mobile but hydrolytically non/barely-active enzyme aggregates form on the surface. At 08:44 min, a part of the fiber becomes mobile and is subsequently removed by the AFM tip.

The formation of clusters that move cohesively while depolymerizing multilayered microfibrils (see Movie S2 for comparison) is not observed. Images are taken from Movie S3. Scale bar and false color scale are included in the figure.

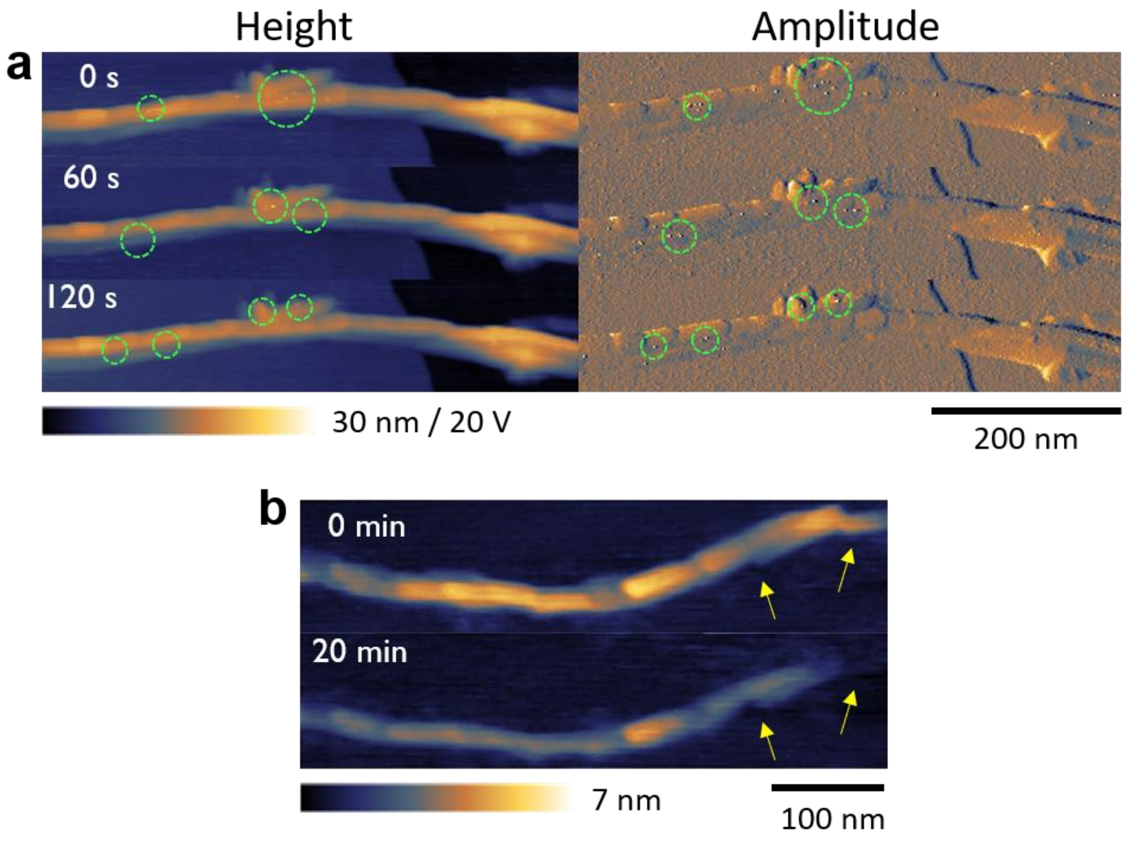

Figure S4. Degradation of cellulose using isolated Cel7B. (a) Height (left) and amplitude (right) images from AFM observation of isolated Cel7B molecules (green circle) on a cellulose fiber. Note, that Cel7B is easier to recognize in the amplitude image. (**b)** Degradation occurs on the entire fiber surface, however, in a more localized manner than with Cel7A. No processive movement of individual Cel7B molecules or cluster formation was observed. Two regions of detectable degradation are highlighted (arrows). Scale bar and false color scale are included in the figure.

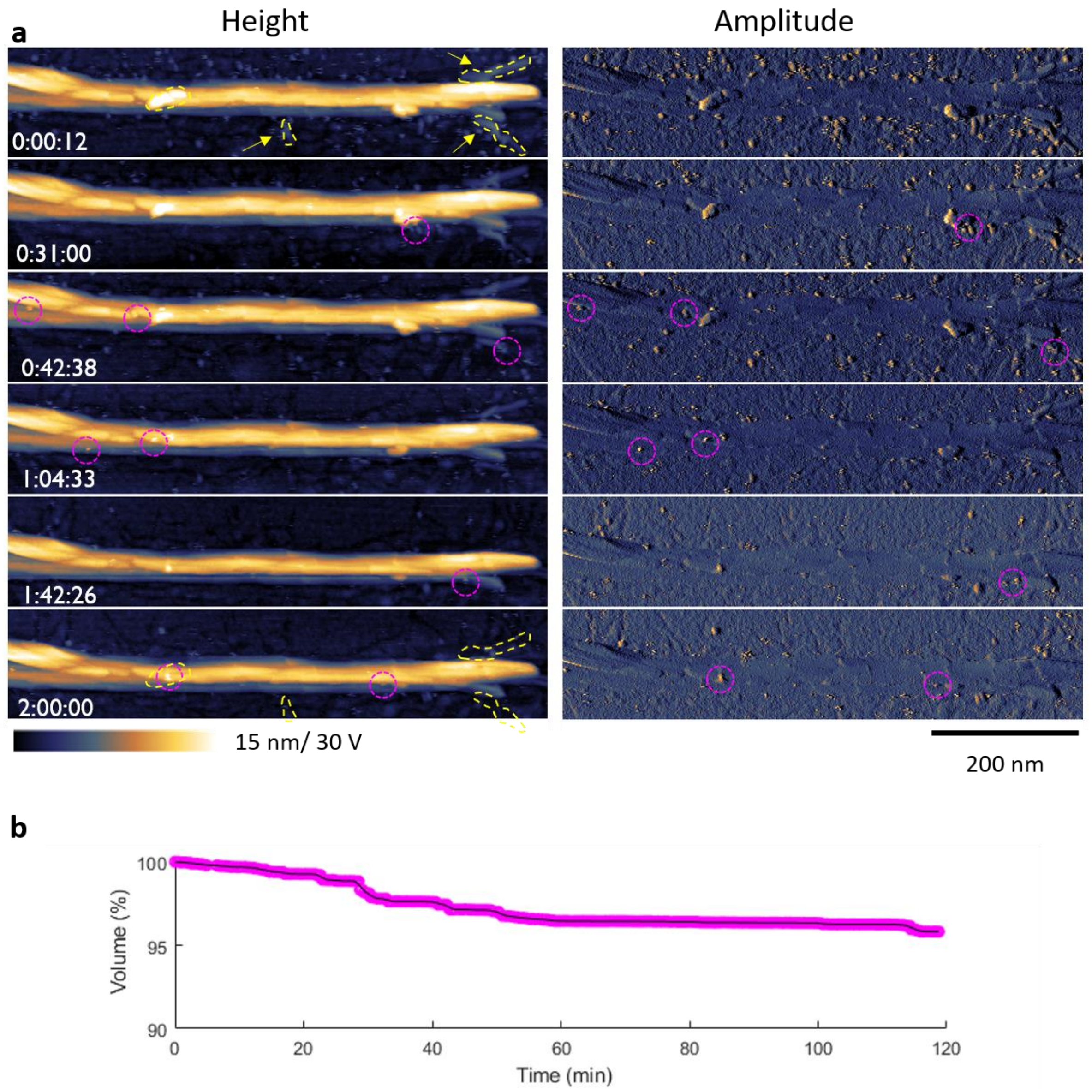

Figure S5. Degradation of cellulose pretreated with Cel7B by Cel7A. (a) Real-time AFM observation of the effect of Cel7A on cellulose pretreated with Cel7B. Multiple Cel7A molecules (magenta circles) are seen in the height (left) and amplitude (right) images. Cellulose fibrils removed within the observed time period are enveloped (yellow) in the first and last image. No cluster was observed in the experiment. **(b)** Volume loss of the fiber over time. After 2 h approximately 4% of the total fiber volume was degraded in a manner similar

to that observed in the absence of Cel7B on untreated fibers (Fig. **S2**). After 50 min the degradation slowed down markedly.

Scale bar and false color scale are included in the figure.

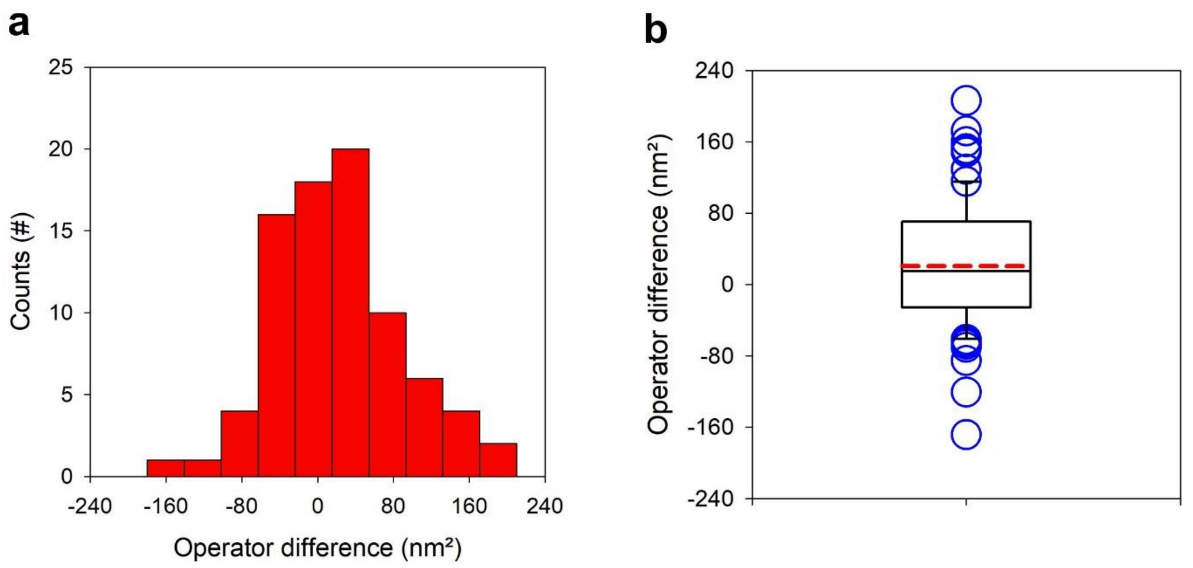

Figure S6. Analysis of the user-induced bias in cluster area measurements. (**a, b)** Difference between two independent users evaluating the area occupied by enzyme clusters, shown as histogram, (**a)**, and boxplot, (**b)**. The median is indicated by a black line, while the mean is shown in red. Outliers are indicated by blue dots.

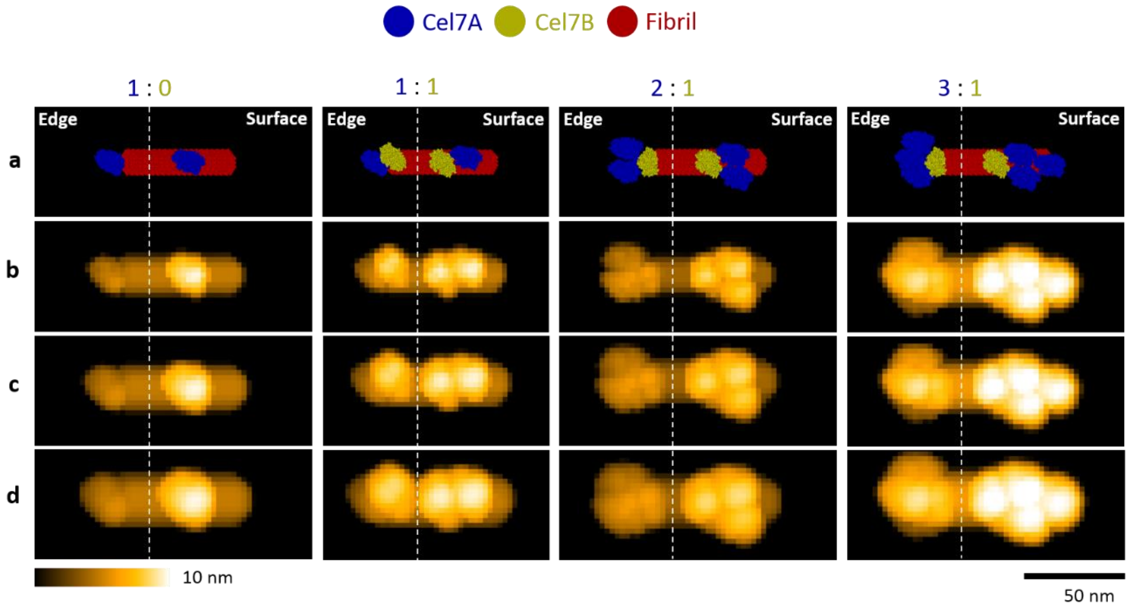

Figure S7. In-silico AFM measurements of different cluster configurations. **(a)** Selection of possible cluster configurations consisting of 1 to 3 Cel7A molecules (blue) and 0 to 1 Cel7B molecules (yellow). All configurations were placed on a cellulose fibril consisting of 36 chains (red). The configurations were placed on both the fibril edge (left) and the fibril surface (right) to account for preferred formation sites (loose ends and kinks). **(b-d)** In silico measurement based on the configurations in **a** with tip radii of 1 nm, 2 nm, and 3 nm, respectively.

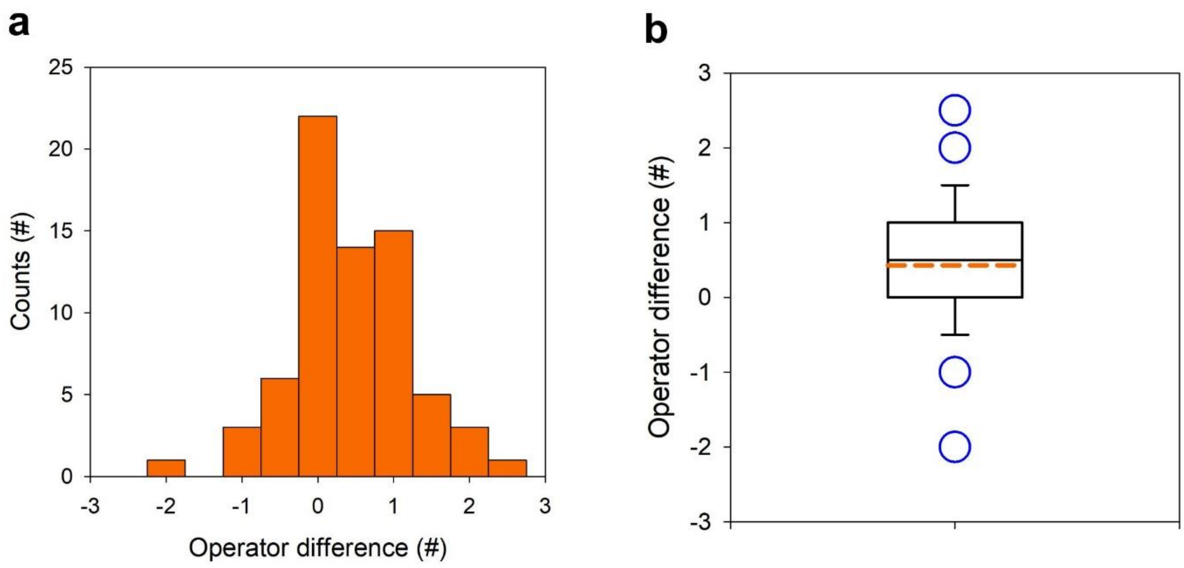

Figure S8. Analysis of the user-induced bias in counting the number of enzymes in the clusters. (a, b) Difference between two independent users in counting the number of enzymes involved in each cluster, shown as histogram (**a)** and boxplot (**b)**. The median is indicated by a black line, while the mean is shown in red. Outliers are indicated by blue dots.

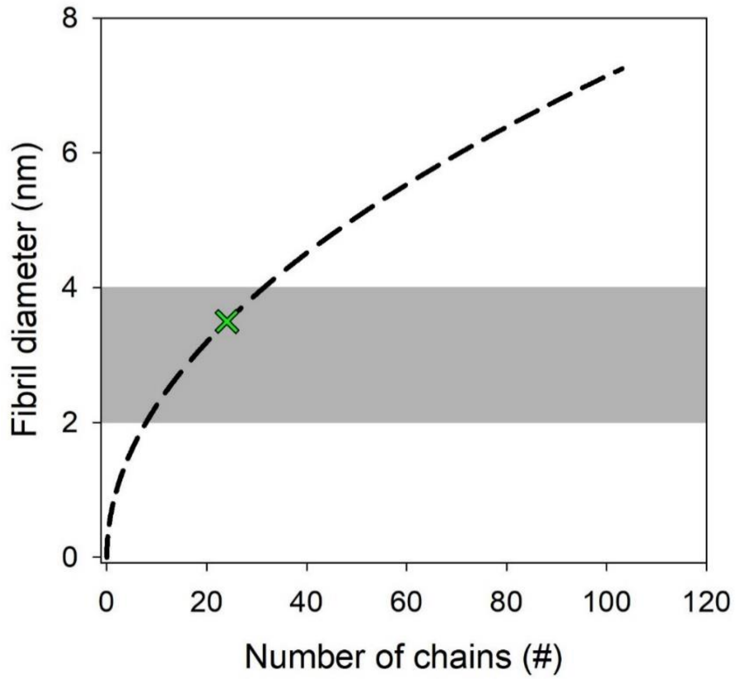

Figure S9. Calculated number of cellulose chains that fit into rod-shaped cellulose fibrils with different diameters. A cylindrical shape was assumed as the geometric representation of the microfibril. The theoretical number expected to fit into each fibril was calculated by dividing various fibril cross-sectional areas resulting from different cylinder diameters by the known cross-section of cellobiose. The measured fibril heights ranged from 2 to 4 nm (highlight in grey) corresponding to 8 to 31 cellulose chains, with an average of 3.5 nm (green x), or 24 chains.

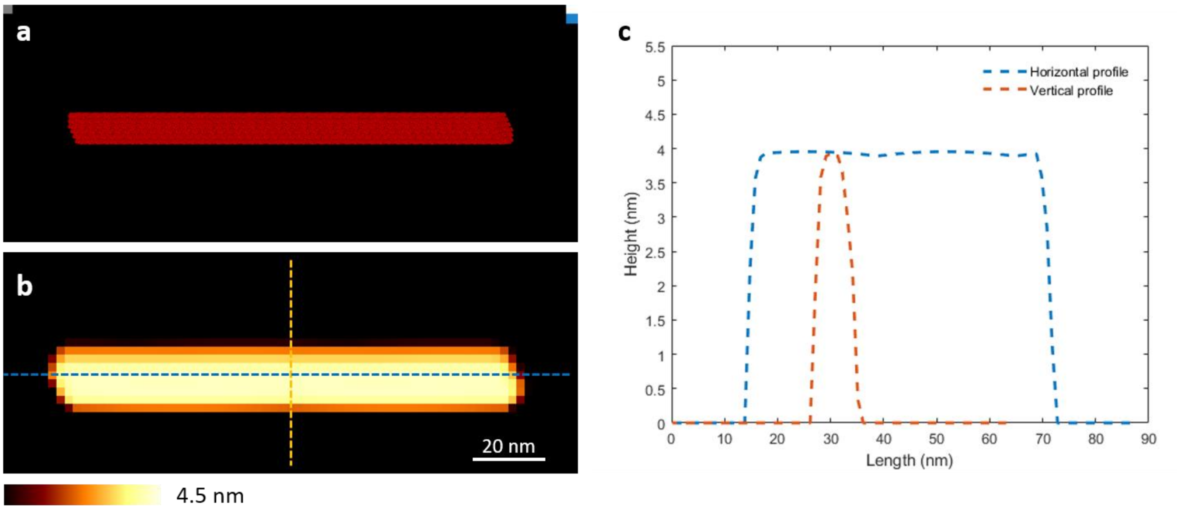

Figure S10. Dimensional properties of a simulated cellulose fibril. (**a)** Imported PDB file of a microfibril with 36 chains and 50 cellobiose units. (**b)** In silico AFM height image based on the structure in (**a**). (**c)** Resulting height profiles in horizontal and vertical direction, indicating, that 36 chains should result in an approximate height of 4 nm.

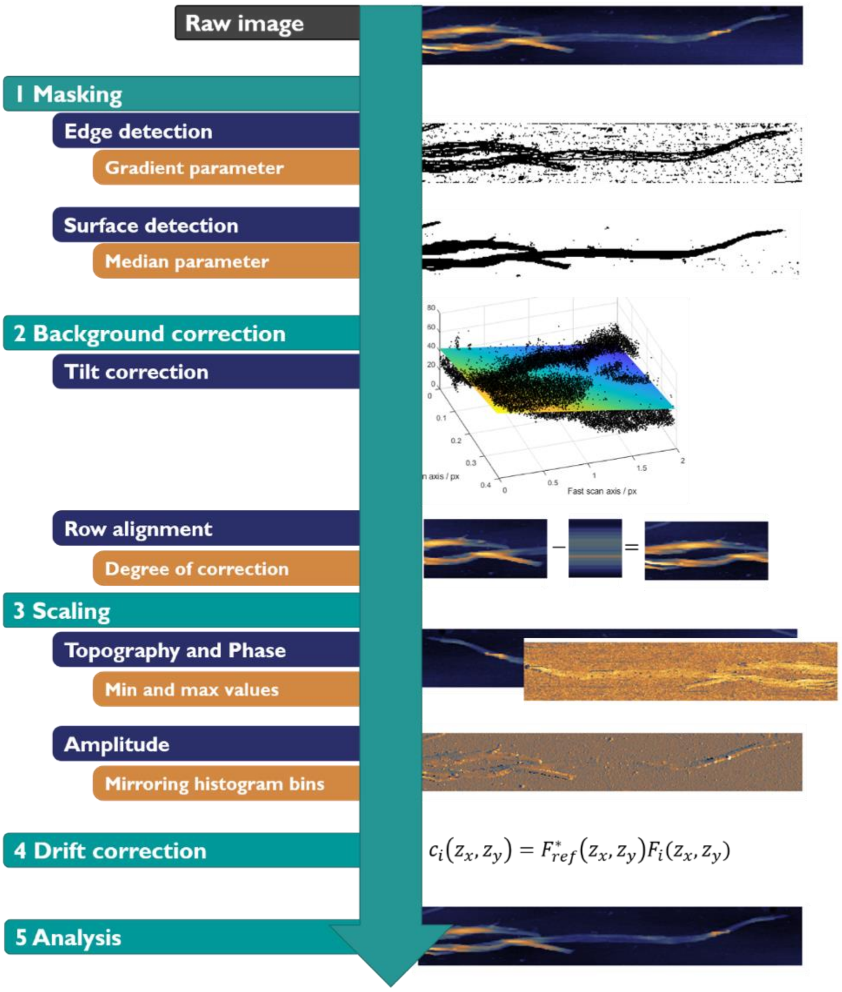

Figure S11. Flowchart summarizing the steps of the image processing procedure performed by the in-house developed MATLAB routine. After importing the raw AFM data, the pixels of the height channel are divided into either object or background pixels (*Masking*). For this purpose, both the edges and the surface of the object are detected. In the next step (*Background correction*) the obtained mask is used to identify and correct the background. The tilt is corrected by fitting a plane through all background pixels and subtracting it. Row alignment is done by fitting linear functions through each horizontal line. Scaling is varying for different data channels: Height and phase channel images within a sequence are scaled to a user set maximum, whereas amplitude channel data is automatically processed to improve visibility of object features (*Scaling*). The drift between preprocessed images within a sequence is corrected using the cross-correlation method (*Drift correction*). The resulting image sequence can be used for further calculations and video generation (*Analysis*).

Supplementary Movie captions

Movie S1: The dispersed cellulases from *Trichoderma reesei* employing two different modes of action during cellulose deconstruction. The complete cellulase mixture of *T. reesei* was used. 100 µL of buffer solution containing the enzymes (15 µg/mL) were added. Individual cellulases, most probably Cel7A, are seen to thin the microfibrils at different surface sites (white dotted circles). Furthermore, instances of rapid deconstruction of a microfibril section were observed (white rectangles).
An area of 250 × 49 / 150 × 30 nm was scanned with 1 frames/s. The step size was chosen to be 1 nm/pixel. Scale bar, time stamps and false color scale are included in the video.

Movie S2: Formation of transient clusters of dispersed cellulase during degradation of bacterial cellulose microfibrils. Real-time observation of the degradation of bacterial cellulose by the addition of 20 µL buffer solution containing the complete cellulase mixture (15 µg/mL). For the first 2 sequences, the image acquisition rate and resolution were 7-8 frames/min and 1 nm/pixel, respectively. Prominent examples of rapid and massive volumetric degradation of fiber parts by transient cellulase clusters are shown. The last sequence was acquired with 1.7 frames/s, showing a fibril fragment seemingly being composed of three segments. The degradation of the first segment starts after the 2 min mark. The second segment follows 1 min later and the last part is attacked after another min.
Scale bar, time stamps and false color scale are included in the video.

Movie S3: Overloading of Cel7A does not result in enzyme clustering behavior. Real-time observation of the degradation of bacterial cellulose by the addition of 100 µL buffer solution containing Cel7A (2 g/L). A scanning area of 100 × 180 nm was observed with a USC-F1.2-k0.15 (NanoandMore GmbH, Wetzlar, Germany) probe. Mostly individual Cel7A molecules can be observed sliding along the fibril surface, however, at certain instances (e.g., 02:22 and 04:00 min mark) larger and mobile, but, hydrolytically non/barely-active enzyme aggregates are observed on the surface. Formation of clusters that show multilayer fibril deconstruction (see Movie S2 for comparison) is not observed. Fiber removal at the 07:00 min mark originates from the fast-scanning carbon AFM tip.

Image acquisition rate and resolution were 0.2 frames/s and 2 nm/pixel, respectively. Scale bar, time stamps and false color scale are included in the video.

Movie S4: Degradation of cellulose fibrils by Cel7A in the absence and presence of Cel7B. Real-time observation of the degradation of bacterial cellulose by the addition of 20 µL buffer solution containing Cel7A (15 µg/mL), which was added at time zero. After 2 h, 20 µL buffer solution containing Cel7B (15 µg/mL) was added. A scanning area of 500 × 100 nm was observed. Magenta dotted circles indicate Cel7A cellulases, white dotted circles indicate clusters. The degradation pattern of the Cel7A acting alone differs drastically from the degradation pattern by Cel7A acting synergistically together with Cel7B. The Cel7A alone does not form enzyme clusters and whole multilayer fibril deconstruction is absent. At the 19 min mark after the addition of Cel7B, a prominent cluster-degradation event can be seen, where the cluster forms at the end of the fibril and slides longitudinally along it while degrading the whole elementary fibril section.

Image acquisition rate and resolution were 5 frames/min and 1 nm/pixel, respectively. Scale bar, time stamps and false color scale are included in the video.

References

(1) Zajki-Zechmeister, K.; Kaira, G. S. S.; Eibinger, M.; Seelich, K.; Nidetzky, B. Processive Enzymes Kept on a Leash: How Cellulase Activity in Multienzyme Complexes Directs Nanoscale Deconstruction of Cellulose. *ACS Catal.* **2021**, *11*, 13530–13542.

(2) Bischof, R. H.; Ramoni, J.; Seiboth, B. Cellulases and beyond: The First 70 Years of the Enzyme Producer *Trichoderma Reesei*. *Microb. Cell Fact.* **2016**, *15*, 106.

(3) Eibinger, M.; Bubner, P.; Ganner, T.; Plank, H.; Nidetzky, B. Surface Structural Dynamics of Enzymatic Cellulose Degradation, Revealed by Combined Kinetic and Atomic Force Microscopy Studies. *FEBS J.* **2014**, *281*, 275–290.

(4) Resch, M. G.; Donohoe, B. S.; Baker, J. O.; Decker, S. R.; Bayer, E. A.; Beckham, G. T.; Himmel, M. E. Fungal Cellulases and Complexed Cellulosomal Enzymes Exhibit Synergistic Mechanisms in Cellulose Deconstruction. *Energy Environ. Sci.* **2013**, *6*, 1858.

(5) Eibinger, M.; Sattelkow, J.; Ganner, T.; Plank, H.; Nidetzky, B. Single-Molecule Study of Oxidative Enzymatic Deconstruction of Cellulose. *Nat. Commun.* **2017**, *8*, 894.

(6) Nečas, D.; Klapetek, P. Gwyddion: An Open-Source Software for SPM Data Analysis. *Open Phys.* **2012**, *10*, 181–188.

(7) Amyot, R.; Flechsig, H. BioAFMviewer: An Interactive Interface for Simulated AFM Scanning of Biomolecular Structures and Dynamics. *PLOS Comput. Biol.* **2020**, *16*, e1008444.

(8) Gomes, T. C. F.; Skaf, M. S. Cellulose-Builder: A Toolkit for Building Crystalline Structures of Cellulose. *J. Comput. Chem.* **2012**, *33*, 1338–1346.

(9) Moon, R. J.; Martini, A.; Nairn, J.; Simonsen, J.; Youngblood, J. Cellulose Nanomaterials Review: Structure, Properties and Nanocomposites. *Chem. Soc. Rev.* **2011**, *40*, 3941–3994.

(10) Usov, I.; Nyström, G.; Adamcik, J.; Handschin, S.; Schütz, C.; Fall, A.; Bergström, L.; Mezzenga, R. Understanding Nanocellulose Chirality and Structure–Properties

Relationship at the Single Fibril Level. *Nat. Commun.* **2015**, *6*, 7564.

(11) O'Dell, P. J.; Mudinoor, A. R.; Parikh, S. J.; Jeoh, T. The Effect of Fibril Length and Architecture on the Accessibility of Reducing Ends of Cellulose Iα to *Trichoderma Reesei* Cel7A. *Cellulose* **2015**, *22*, 1697–1713.

(12) Nishiyama, Y.; Sugiyama, J.; Chanzy, H.; Langan, P. Crystal Structure and Hydrogen Bonding System in Cellulose I(Alpha) from Synchrotron X-Ray and Neutron Fiber Diffraction. *J. Am. Chem. Soc.* **2003**, *125*, 14300–14306.

(13) *Cellulose Chemistry and Properties: Fibers, Nanocelluloses and Advanced Materials*; Rojas, O. J., Ed.; Advances in Polymer Science; Springer International Publishing: Cham, 2016.

(14) Sugar, J. D.; Cummings, A. W.; Jacobs, B. W.; Robinson, D. B. A Free MATLAB Script for Spatial Drift Correction. *Micros. Today* **2014**, *22*, 40–47.
